# Incidence of SARS-CoV-2 infection in hospital workers before and after vaccination programme in East Java, Indonesia – a retrospective cohort study

**DOI:** 10.1016/j.lansea.2022.100130

**Published:** 2022-12-12

**Authors:** Gatot Soegiarto, Dewajani Purnomosari, Laksmi Wulandari, Bagus Aulia Mahdi, Karin Dhia Fahmita, Satrio Tri Hadmoko, Hendra Ikhwan Gautama, Muhammad Edwin Prasetyo, Dewi Prasetyaningtyas, Pujo Prawiro Negoro, Nur Arafah, Cita Rosita Sigit Prakoeswa, Anang Endaryanto, Desak Gede Agung Suprabawati, Damayanti Tinduh, Eka Basuki Rachmad, Erwin Astha Triyono, Joni Wahyuhadi, Catur Budi Keswardiono, Feby Elyana Wardani, Fitriyah Mayorita, Nunuk Kristiani, Ari Baskoro, Deasy Fetarayani, Wita Kartika Nurani, Delvac Oceandy

**Affiliations:** a Division of Allergy and Clinical Immunology, Department of Internal Medicine, Faculty of Medicine, Universitas Airlangga - Dr. Soetomo General Academic Hospital, Surabaya, Indonesia; b Department of Histology and Cell Biology, Faculty of Medicine Public Health and Nursing, Universitas Gadjah Mada, Yogyakarta, Indonesia; c Department of Pulmonology and Respiratory Medicine, Faculty of Medicine, Universitas Airlangga - Dr. Soetomo General Academic Hospital, Surabaya, Indonesia; d Department of Internal Medicine, Faculty of Medicine, Universitas Airlangga - Dr. Soetomo General Academic Hospital, Surabaya, Indonesia; e Department of Dermatology and Venereology, Faculty of Medicine, Universitas Airlangga - Dr. Soetomo General Academic Hospital, Surabaya, Indonesia; f Department of Child Health, Faculty of Medicine, Universitas Airlangga - Dr. Soetomo General Academic Hospital, Surabaya, Indonesia; g Division of Oncology, Department of Surgery, Faculty of Medicine, Universitas Airlangga - Dr. Soetomo General Academic Hospital, Surabaya, Indonesia; h Department of Physical Medicine and Rehabilitation, Faculty of Medicine, Universitas Airlangga - Dr. Soetomo General Academic Hospital, Surabaya, Indonesia; i Medical Service Bureau, Dr. Soetomo General Academic Hospital, Surabaya, Indonesia; j Division of Tropical Disease and Infection, Department of Internal Medicine, Faculty of Medicine, Universitas Airlangga - Dr. Soetomo General Academic Hospital, Surabaya, Indonesia; k Department of Neurosurgery, Faculty of Medicine, Universitas Airlangga – Dr. Soetomo General Academic Hospital, Surabaya, Indonesia; l Syarifah Ambami Rato Ebu Hospital, Bangkalan, Madura, East Java, Indonesia; m Division of Cardiovascular Sciences Faculty of Biology Medicine and Health, University of Manchester, Manchester Academic Health Science Centre, Manchester, United Kingdom; n Department of Biomedical Science, Faculty of Medicine, Universitas Airlangga, Surabaya, Indonesia

**Keywords:** COVID-19, Healthcare workers, Vaccine, Hospitalisation, Co-morbidity

## Abstract

**Background:**

The incidence of the Coronavirus Disease 2019 (COVID-19) among healthcare workers (HCWs) is widespread. It is important to understand COVID-19 characteristics among HCWs before and after vaccination. We evaluated the incidence of COVID-19 among HCWs in East Java, Indonesia comparing the characteristics of the disease between the pre- vs post-vaccination periods.

**Methods:**

A retrospective observational study was conducted among HCWs in two major hospitals in East Java, Indonesia, between April 01, 2020, and Oct 31, 2021. All HCWs were offered vaccination with inactivated viral vaccine (CoronaVac) from Jan 15, 2021. Therefore, we divided the time of the study into the pre-vaccination period (between April 01, 2020, and Jan 14, 2021) and post-vaccination period (between Jan 15 and Oct 31, 2021). We then compared the pattern of COVID-19 infections, and hospitalisations between these periods.

**Findings:**

A total of 434 (15.1%) and 649 (22.6%) SARS-CoV-2 infections were reported among study participants (n = 2878) during the pre-vaccination and post-vaccination periods, respectively. The vaccine effectiveness was 73.3% during the first 3–4 months after vaccination but this decreased to 17.6% at 6–7 months after vaccination, which coincided with the emergence of the delta variant. The overall hospitalisation rate was reduced from 23.5% in the pre-vaccination period to 14.3% in the post-vaccination period. Hypertension appeared to be the strongest risk factor affecting hospitalisation in the pre-vaccination period. However, the risk due to hypertension was reduced in the post-vaccination period.

**Interpretation:**

The risk to contract COVID-19 remains high among HCWs in East Java, Indonesia. Vaccination is important to reduce infection and hospitalisation. It is essentially important to evaluate the characteristics of COVID-19 infection, hospitalisation, the impact of co-morbidities and vaccine effectiveness in order to improve the measures applied in protecting HCWs during the pandemic.

**Funding:**

Mandate Research Grant No:1043/UN3.15/PT/2021, 10.13039/501100008463Universitas Airlangga, Indonesia.

**Research in context**

**Evidence before this study**

Several studies have reported a relatively higher incidence of COVID-19 in healthcare workers (HCWs) compared to the wider population. However, there is only limited information comparing the characteristics of COVID-19 cases, hospitalisation, the effects of vaccination and the presence of co-morbidities between the periods before the vaccination programme started and after the vaccination programme was implemented in HCWs.

**Added value of this study**

The 30-day incidence of COVID-19 among HCWs in two major hospitals in East Java increased from 1.57% before the start of the vaccination to 2.34% after the vaccination programme. The increased incidence of COVID-19 could be attributed to the emergence of the delta variant. In comparison, the vaccine effectiveness in protecting against SARS-CoV-2 infection in HCWs was around 73% before the emergence of the delta variant but decreased to 17.6% at the period when the delta variant became dominant. However, there was a reduction in the rate of hospitalisation from 23.5% in the pre-vaccination period to 14.3% in the post-vaccination period. Our data adds new knowledge regarding the different pattern of COVID-19 cases and hospitalisation among HCWs before and after vaccination as well as the efficacy of vaccination using inactivated virus vaccine in HCWs.

**Implications of all the available evidence**

Our data suggested that the risk of contracting COVID-19 among HCWs in East Java, Indonesia remains high, both before and after the vaccination programme. Although vaccination is important to reduce the rate of infection and hospitalisation, its effectiveness could decline overtime and during the emergence of new virus variants. Effective measures to protect HCWs from contracting COVID-19 always need to be reviewed and strictly implemented during the pandemic and beyond.

## Introduction

In addition to therapeutic medications, vaccines and rapid diagnostics have been regarded as key tools to overcome the Coronavirus Disease 2019 (COVID-19) pandemic.^1^ Several types of COVID-19 vaccines have successfully been developed and some of them have shown strong effectiveness in preventing SARS-CoV-2 infection or reducing the severity of the disease. One of the most widely used COVID-19 vaccines is the inactivated viral vaccine. The clinical trials and real-life data shows that inactivated viral vaccines provides significant level of protection against severe COVID-19.^2^^,^^3^ Although there were fewer reports regarding the effectiveness compared to the mRNA or adenoviral vaccine, the inactivated viral vaccine has been approved and used in as many as 91 countries.^4^

Indonesia is among the first countries in the world which started a mass vaccination programme at the beginning of 2021.^5^ Following a phase 3 clinical trial in the country,^6^ the Indonesian authority for drug and food approval (BPOM) authorised the emergency use of inactivated SARS-CoV-2 vaccine (CoronaVac) in the national vaccination programme, which was started on Jan 13, 2021. Healthcare workers (HCWs) were the first group of people who received the COVID-19 vaccine during that period.

Despite the high protection of COVID-19 vaccines as reported in the phase 3 trials and in the initial real world data, there were concerns about reduction in vaccine effectiveness over time; possibly due to the waning of immunity^7^^,^^8^ and the emergence of new virus variants.^9^^,^^10^ Therefore, it is important to understand whether COVID-19 vaccination programme affected the pattern and characteristics of COVID-19 incidence and hospitalisation in the population. Since HCWs have a greater risk for SARS-CoV-2 infection, the surveillance data to record COVID-19 incidence and hospitalisation are important to provide stakeholders and policy makers with information to consider the next strategies to protect HCWs from COVID-19. In this study a retrospective analysis was conducted on HCWs in East Java, Indonesia, who were offered inactivated SARS-CoV-2 vaccines at the early stage of the national COVID-19 vaccination programme. The main objective was to evaluate the incidence of COVID-19 in a cohort of HCWs during the pre-vaccination and post-vaccination periods. We also compared the rate of hospitalisation between these two periods and analysed whether demographic characteristics and the presence of co-morbidity affected the pattern of infections and hospitalisations in these two periods of the study.

## Methods

### Study participants

A cohort of 2878 HCWs agreed to participate in this retrospective observational study. The participants were HCWs from two major hospitals in East Java, Indonesia: Dr. Soetomo General Academic Hospital in Surabaya (1805 participants) and Syarifah Ambami Rato Ebu Hospital in Bangkalan (1073 participants). The total number HCWs in both hospitals at the time of the study was 7311. Thus, the participants were 39.4% of the total HCWs in both hospitals. All the HCWs in these hospitals were offered two doses of inactivated SARS-CoV-2 vaccine (CoronaVac). Based on the power calculation method described by Lwanga and Lemeshow^11^ a minimum of 1537 samples was required to estimate incidence rate with relative precision of 5% and 95% CI. Our sample size exceeded this requirement.

### Data collection

**Fig. 1:**
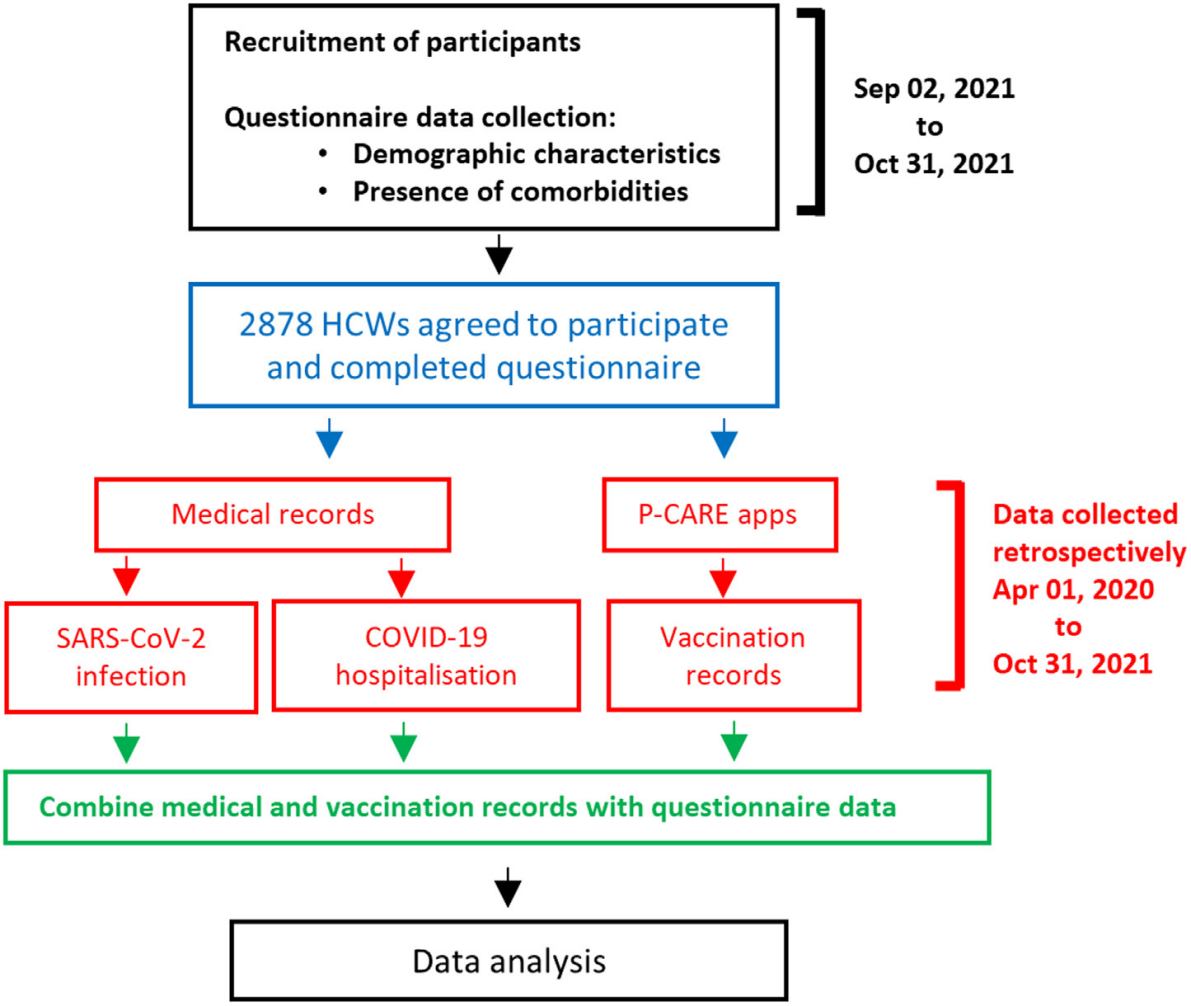
**Study workflow.** The study cohort involving health care workers (HCWs) in Dr Soetomo General Hospital, Surabaya and Syarifah Ambami Rato Ebu, Bangkalan. Both hospitals are located in East Java province, Indonesia. Participants were recruited and baseline demographic and comorbidities data were collected between Sept 02 and Oct 31, 2021. Medical records and vaccination data were collected retrospectively spanning the period between April 01, 2020 and Oct 31, 2021.

Data on demographic characteristics and the presence of comorbidities were collected using a computer-based questionnaire. The participants were recruited, and the questionnaires were completed between Sept 2 and Oct 31, 2021. We then conducted retrospective observations using hospital medical records to evaluate the incidence of SARS-CoV-2 infections in the study participants which occurred between April 01, 2020, and Oct 31, 2021. We also used the medical records to assess hospitalisation due to COVID-19 during the period above. We collected vaccination records of the participants from the P-CARE application, which was used by the health authority to record and monitor the implementation of the national vaccination programme in Indonesia. We combined data from the medical and vaccination records with the data from the questionnaire and tabulated them in SPSS software version 25 (IBM Corp., Armonk, NY). The workflow of this study is presented in Fig. 1.

COVID-19 infection that occurred at more than 14 days after the second dose of vaccination was categorised as breakthrough infection (infection in a fully vaccinated participant). Otherwise, it was categorised as infection in incomplete or unvaccinated individuals.

Analysis of SARS-CoV-2 variants in East Java, Indonesia was performed using data from GISAID.^12^ The sequence metadata of viruses isolated from East Java, Indonesia during the study period (between April 01, 2020, and Oct 31, 2021) were retrieved from the GISAID website and were analysed to evaluate the prevalence of SARS-CoV-2 variants in this region. The list of virus sequence entries which were used in this study can be accessed in the supplementary material.

### Analysis of vaccine effectiveness

Analysis of vaccine effectiveness was conducted using the following equation as described in previous publication^13^:

$$\text{Vaccine}\;\text{effectiveness}=\frac{\text{Incidence}\;\text{in}\;\text{unvaccinated}\;\text{participants}-\text{incidence}\;\text{in}\;\text{vaccinated}\;\text{participants}}{\text{Incidence}\;\text{in}\;\text{unvaccinated}\;\text{participants}}\times 100\%$$

### Ethics statement

We received ethical approval from the Local Health Research Ethics Committee of Dr Soetomo General Academic Hospital, Surabaya, Indonesia (No. 0145/KEPK/II/2021) to conduct this study. All study participants signed the informed consent form to confirm their agreement to participate in this study.

### Data analysis

We used IBM SPSS Statistics software version 25 (IBM Corp., Armonk, NY) to analyse the data. To analyse categorical data, we used chi-squared or Fisher's exact test if it is a 2 × 2 crosstab. The incidence of SARS-CoV-2 infection was analysed according to the period of study, i.e. pre-vaccination period (between April 01, 2020, and Jan 14, 2021) and post-vaccination period (between Jan 15 and Oct 31, 2021). We used chi-squared or Fisher's exact tests to determine if the incidence of infection was correlated with demographic characteristics (i.e. sex, age, and work unit) and to determine if hospitalisation was associated with the presence of comorbidities. Multivariate logistic regression analysis was conducted to determine the odds ratio (OR) of being hospitalised by adjusting for the confounding effects of each variable. Age, sex, body mass index (BMI), history of hypertension, diabetes mellitus, cardiovascular diseases and lung diseases/asthma were included in the multivariate analysis. This was based on previous findings showing that these variables were significantly associated with the severity of COVID-19.^14^, ^15^, ^16^ A *P* value < 0.05 was considered statistically significant.

### Role of the funding source

This study was funded by the Mandate Research Grant No: 1043/UN3.15/PT/2021 from 10.13039/501100008463Universitas Airlangga, Surabaya. The funder did not have any role in the study design, data acquisition and analysis, data interpretation, or manuscript writing.

## Results

### Study participants

We conducted this retrospective study to assess the features of COVID-19 infection amongst HCWs in two major hospitals in East Java Indonesia between April 01, 2020, and Oct 31, 2021. The national vaccination programme in Indonesia was initiated on Jan 13, 2021 and the first HCWs in East Java received the COVID-19 vaccine on Jan 15, 2021, so we divided the study period into two categories: pre-vaccination period (between April 01, 2020, and Jan 14, 2021) and post-vaccination period (between Jan 15 and Oct 31, 2021).

Data were collected from a total of 2878 participants. Most of the study participants were medical staffs (including physicians, residents, and medical students; 25.7%) and nurses (27.7%), whereas 19.5% of participants were administrative staff members. Technical staff (including radiographers, hospital information technology personnel, electromedical engineers or technician, hospital infrastructure and facilities maintenance personnel) accounted for 11.4% of participants and 2.5% of participants are pharmacists (Table 1).

**Table 1:** Demographic data of study participants.

	Study participants (n = 2878)
Gender	
Male	1515 (52.6%)
Female	1363 (47.4%)
Age (mean ± SD) (years)	36.15 ± 9.88
Age group (years)	
< 30	787 (27.3%)
30–39	1202 (41.8%)
40–49	504 (17.5%)
≥50	385 (13.4%)
Work unit	
Medical staff (physician, resident, medical student)	739 (25.7%)
Nurse	796 (27.7%)
Pharmacist	71 (2.5%)
Technical staff	329 (11.4%)
Administrative staff	560 (19.5%)
Others (security, cleaning service, drivers, social care workers)	383 (13.3%)

Demographic characteristics of the study participants are described in Table 1. The average age was 36.15 ± 9.88 years with most of the participants (41.8%) were in the 30–39 years age group. The distribution of sex was slightly higher in males (52.6%).

**Fig. 2:**
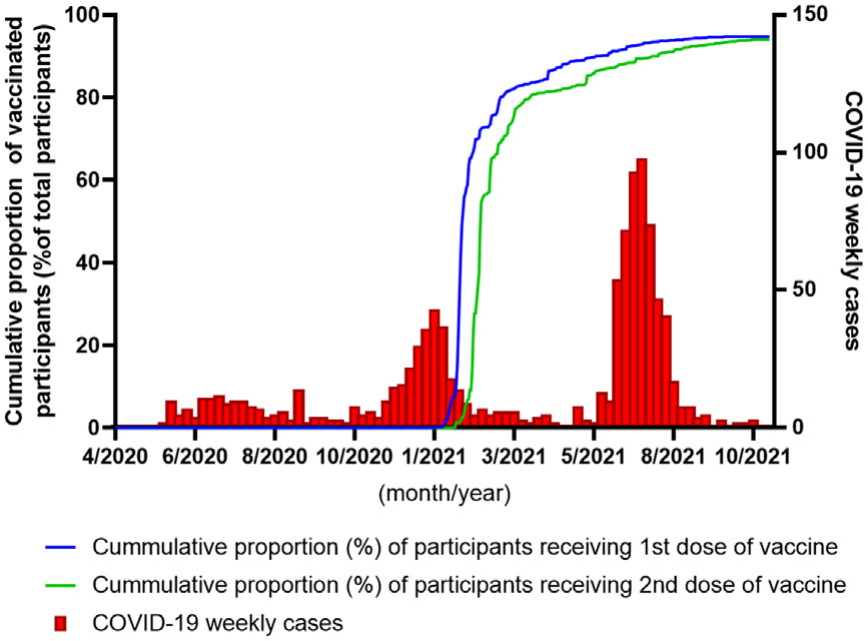
**COVID-19 weekly cases and vaccination coverage among the study participants.** The weekly incidence of COVID-19 cases among healthcare workers in two major hospitals in East Java between April 01, 2020 and Oct 31, 2021. There were two surges of COVID-19 cases, first in December 2020–January 2021 and second in June–July 2021. The incidence reflected the national and regional epidemic curve as described elsewhere.^17^^,^^18^ The vaccination program started in mid-January 2021 and by April 2021, 82% of participants had received full doses of vaccination.

The uptake of the COVID-19 vaccination amongst study participants is shown in Fig. 2. The first dose of vaccine was administered to HCWs starting on Jan 15, 2021, whereas the second dose of vaccine was given two weeks after the first dose. All participants received CoronaVac (inactivated SARS-CoV-2 vaccine) since this was the only type of vaccine used by the Indonesian Government at that time. As shown in Fig. 2 by the start of March, 2021, about 76% of the participants had received the first dose of vaccine and around 66% of them had received the second dose. By the end of April, 2021, as many as 88% participants had received the first dose and 82% had received the second dose. At the end of the observation (Oct 31,2021), 93% of participants had received two doses of the vaccine. This indicates a very high uptake of COVID-19 vaccination amongst HCWs in major hospitals in East Java.

### Incidence of SARS-CoV-2 infection

The epidemic curve of COVID-19 incidence amongst study participants during the period of this study is described in Fig. 2, showing two waves of COVID-19 surges amongst HCWs with the weekly cases peaking for the first time at the end of the period between December, 2020 and January, 2021; and peaking for the second time during the period between June and July 2021.

It was likely that the dominant virus strain, which circulated among HCWs in these hospitals, was similar to the virus strain which circulated in the wider population in East Java at the respective time. Therefore, we examined virus sequence data from GISAID database to understand the dominant SARS-CoV-2 strains that circulated in East Java during the period of this study. As shown in Supplementary Fig. S1 there were two different types of virus strains that were dominant in East Java. The first one was the lineage B.1.470. This variant was dominant during the first surge of COVID-19 infection between December 2020 and March 2021. The second dominant strain was the Delta variant (Pango lineage B.1.617.2). This variant was dominant during the second surge between June 2021 and August 2021. Please see Supplementary Table S1 for detailed information on virus data included in this analysis.

### Characteristics of infected participants

The characteristics of infected participants are described in Table 2. During the pre-vaccination period (289 days), the cumulative incidence of COVID-19 was 434 (15.1%) cases. This corresponded to a 30-day incidence of 1.57%. On the other hand, during the post vaccination period (290 days), the cumulative incidence was higher (649 cases, 22.6%) which was equal to a 30-day incidence of 2.34%. In the pre-vaccination period, the incidence of infection seemed to be higher among medical staffs and nurses compared to staff members of other work units, however, we observed an increased incidence of infection among staff of all work units during the post-vaccination period. There were also changes in terms of the age group of infected participants, in which the 30–39 years age group was the highest during the pre-vaccination period, whereas in the post-vaccination period it was the 40–49 years age group that had the highest incidence.

**Table 2:** Incidence of SARS-Cov-2 infection.

	Pre-vaccination period (April 01, 2020–Jan 14, 2021) (n = 2878)	Post-vaccination period (Jan 15, 2021–Oct 31, 2021) (n = 2878)
Cumulative incidence of SARS-CoV-2 infection	434/2878 (15.1%)	649/2878 (22.6%)
Gender		
Male	253/1515 (16.7%)	319/1515 (21.1%)
Female	181/1363 (13.3%)	330/1363 (24.2%)
*P* value (Fisher's exact)	0.011	0.044
Age group (years)		
< 30	100/787 (12.7%)	157/787 (19.9%)
30–39	200/1202 (16.6%)	283/1202 (23.5%)
40–49	71/504 (14.1%)	122/504 (24.2%)
≥ 50	63/385 (16.4%)	87/385 (22.6%)
*P* value (Chi-square)	0.085	0.210
Work unit		
Medical staff	122/739 (16.5%)	145/739 (19.6%)
Nurse	126/796 (15.8%)	203/796 (25.5%)
Pharmacist	6/71 (8.5%)	24/71 (33.8%)
Technician	39/329 (11.9%)	74/329 (22.5%)
Administrative staff	72/560 (12.9%)	117/560 (20.9%)
Others	69/383 (18.0%)	86/383 (22.5%)
*P* value (Chi-square)	0.044	0.018

### Vaccine effectiveness in preventing infection

We then analysed the status of vaccination of participants at the time of infection. During the period of this study, 2676 participants (93% of total subjects) had received two doses of inactivated viral vaccine. Of these, 572 participants (21.4%) had been infected with SARS-CoV-2 virus at >14 days after receiving their second dose of vaccination. In contrast, we observed 77 (38.1%) COVID-19 infections among participants with either incomplete (receiving only one dose) or no vaccination at all, out of a total of 202 subjects in this category. The difference was statistically significant (*P* < 0.0001, Fisher's exact test, Table 3). This finding indicated approximately 44% protection against infection in participants with two doses of inactivated viral vaccine between Jan 15 and Oct 31, 2021.

**Table 3:** Incidence of SARS-CoV-2 infection according to vaccination status at post-vaccination period.

	Status of vaccination at the time of infection		Fisher's Exact test
	Fully vaccinated	Incomplete or unvaccinated	
SARS-CoV-2 infection – Jan 15, 2021 to May 31, 2021 (pre-Delta period)			
Yes	48 (1.94%)	29 (7.25%)	
No	2430 (98.06%)	371 (92.75%)	
Total	2478 (100%)	400 (100%)	*P* < 0.0001
SARS-CoV-2 infection – June 1, 2021 to Oct 31, 2021 (Delta period)			
Yes	524 (19.58%)	48 (23.76%)	
No	2152 (80.42%)	154 (76.24%)	
Total	2676 (100%)	202 (100%)	*P* = 0.1697
SARS-CoV-2 infection (combine)			
Yes	572 (21.4%)	77 (38.1%)	
No	2104 (78.6%)	125 (61.9%)	
Total	2676 (100%)	202 (100%)	*P* < 0.0001

Since vaccine effectiveness might be reduced due to the presence of new virus variants, we compared infection rates between the period when the B.1.470 virus variant was still dominant (January–May 2021) and the period when the Delta variant (B.1.617.2) was dominant (June–October 2021). Our observations showed that the incidence of SARS-CoV-2 infections amongst fully vaccinated participants was lower than incomplete/unvaccinated participants during January–May 2021 and was statistically significant (1.93% vs 7.25%, *P* < 0.0001), indicating a vaccine effectiveness of around 73.3%. In contrast, in the period between Jun–Oct 2021, there were 19.6% incidence of breakthrough infection compared to 23.8% infection amongst unvaccinated individuals (*P* = 0.1697) with the vaccine effectiveness markedly reduced to 17.6% (Table 3).

### Incidence and characteristics of hospitalised patients

We analysed the number of hospitalised subjects due to COVID-19. Although there was an increase in the total number of hospitalised participants during the post vaccination period, we observed a reduction in the proportion of hospitalisation/total infected participants from 23.5% during the pre-vaccination period to 14.3% during the post-vaccination period (Table 4). We then assessed the demographic pattern and the presence of co-morbidities of hospitalised participants. As expected, the hospitalised subjects in both periods of study were significantly older and predominantly male (Table 4). We also found that there was higher incidence of hospitalisation in participants with body mass index (BMI) assessed obesity (BMI≥30). When we analysed specific comorbidities, we found a significant trend of higher hospitalisation in subjects with hypertension and diabetes mellitus, however, there was no significant correlation between the presence of cardiovascular and lung diseases with the incidence of hospitalisation (Table 4).

**Table 4:** Demographic and clinical characteristics of hospitalised participants due to COVID-19.

	Hospitalisation (Pre-vaccination period)			Hospitalisation (Post-vaccination period)		
	No (n = 332) (76.5%)	Yes (n = 102) (23.5%)		No (n = 556) (85.7%)	Yes (n = 93) (14.3%)	
Age	35.9 ± 9.03	38.9 ± 10.45	*P* = 0.005^a^	36.12 ± 9.1	38.5 ± 11.47	*P* = 0.028^a^
Sex						
Male	187 (73.9%)	66 (26.1%)		262 (82.1%)	57 (17.9%)	
Female	145 (80.1%)	36 (19.9%)	*P* = 0.138^b^	294 (89.1%)	36 (10.9%)	*P* = 0.013^b^
Comorbidity						
No comorbidity	240 (81.4%)	56 (18.6%)		395 (88%)	54 (12%)	
1 comorbidity	76 (72.4%)	29 (27.6%)		121 (85.2%)	21 (14.8%)	
>1 comorbidities	15 (45.5%)	18 (54.5%)	*P* < 0.0001^c^	40 (69%)	18 (31%)	*P* = 0.001^c^
BMI						
BMI<30	280 (78.2%)	78 (21.8%)		485 (86.6%)	75 (13.4%)	
BMI≥30	51 (68%)	24 (32%)	*P* = 0.072^b^	71 (79.8%)	18 (20.2%)	*P* = 0.102^b^
Hypertension						
No	313 (79%)	83 (21%)		493 (86.8%)	75 (13.2%)	
Yes	19 (50%)	19 (50%)	*P* < 0.0001^b^	63 (77.8%)	18 (22.2%)	*P* = 0.041^b^
History of DM						
No	327 (77.1%)	97 (22.9%)		538 (86.5%)	84 (13.5%)	
Yes	5 (50%)	5 (50%)	*P* = 0.060^b^	18 (66.7%)	9 (33.3%)	*P* = 0.009^b^
CVD History						
No	326 (76.5%)	100 (23.5%)		552 (86%)	90 (14%)	
Yes	6 (75%)	2 (25%)	*P* = 0.92^b^	4 (57.1%)	3 (42.9%)	*P* = 0.065^b^
Lung disease/asthma						
No	310 (77.7%)	89 (22.3%)		514 (86.4%)	81 (13.6%)	
Yes	22 (62.9%)	13 (37.1%)	*P* = 0.06^b^	42 (77.8%)	12 (22.2%)	*P* = 0.102^b^

BMI, body mass index; CVD, cardiovascular disease; DM, diabetes mellitus.

a *t* test.

b Fisher's exact test.

c Chi-square test.

**Fig. 3:**
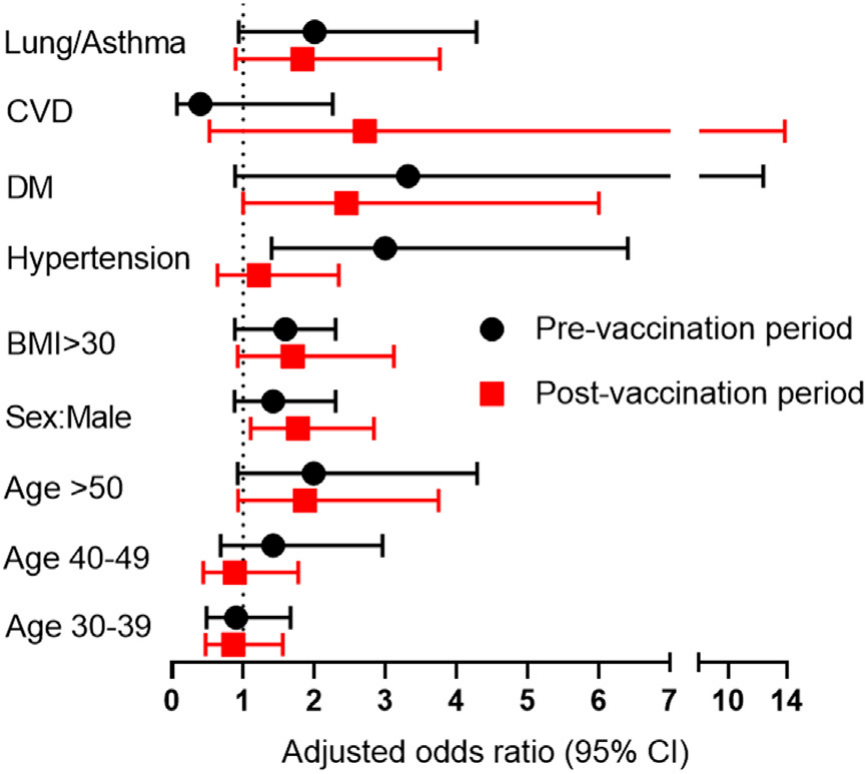
**The adjusted odds ratio of hospitalisation before and after vaccination.** The adjusted odds ratio (aOR) and 95% CI to predict the risk of hospitalisation due to COVID-19 in the presence of the conditions above in comparison with the reference conditions. The reference conditions were: age <30 years, female, BMI<30, and absence of each co-morbidity. The reduction in the risk for hospitalisation was markedly reduced in participants with hypertension after the vaccination programme. CVD: cardiovascular diseases, DM: diabetes mellitus.

We next conducted multivariate analysis to further examine the association between demographic features, co-morbidities, and hospitalisation. As shown in Table 5, history of hypertension appeared to have significant association with hospitalisation during the pre-vaccination period, whereas in the post-vaccination period male participants and those who had diabetes mellitus showed significant association with hospitalisation. Remarkably, when we evaluated the trend of the odds ratio (OR) of hospitalisation before and after the vaccination programme, we observed a marked reduction of OR in participants with history of hypertension whereas in other factors the OR of hospitalisation seemed comparable or only slightly changed (Fig. 3).

**Table 5:** Multivariate analysis of the association between demographic features, co-morbidities and hospitalisation during pre-vaccination and post-vaccination period.

	Pre-vaccination period		Post-vaccination period	
	Adjusted Odds ratio for hospitalisation (95% CI)	*P* value	Adjusted Odds ratio for hospitalisation (95% CI)	*P* value
Age group (<30 as reference)				
30–39	0.901 (0.486–1.669)	0.740	0.860 (0.474–1.560)	0.619
40–49	1.424 (0.685–2.961)	0.344	0.884 (0.440–1.775)	0.729
> 50	1.994 (0.927–4.292)	0.078	1.870 (0.932–3.751)	0.078
Sex male (female as reference)	1.425 (0.882–2.303)	0.148	1.773 (1.108–2.838)	**0.017**
BMI ≥30 (BMI <30 as reference)	1.596 (0.883–2.883)	0.121	1.701 (0.927–3.121)	0.086
HT history (no HT as reference)	2.998 (1.402–6.410)	**0.005**	1.226 (0.640–2.348)	0.539
DM history (no DM as reference)	3.319 (0.888–12.405)	0.075	2.455 (1.004–6.002)	**0.049**
CVD history (no CVD as reference)	0.400 (0.071–2.261)	0.300	2.711 (0.529–13.885)	0.232
Lung disease/asthma history (no lung disease/asthma as reference)	2.005 (0.939–4.284)	0.072	1.836 (0.894–3.767)	0.098
No complete vaccination (complete vaccination as reference)			1.224 (0.616–2.436)	0.564

CVD, cardiovascular disease; DM, diabetes mellitus.

*P* values < 0.05 are printed in bold.

## Discussion

The present study described the pattern of COVID-19 incidence and hospitalisation among HCWs in two major hospitals in East Java, Indonesia, comparing the incidence between the pre-vaccination and post-vaccination period. We found that the cumulative incidence of COVID-19 infection in our cohort during the pre-vaccination period was 15.1%, which was equal to a 30-day incidence of 1.57%, whereas during the post-vaccination period the COVID-19 cumulative incidence was 22.6% (or 2.34% of 30-day incidence). This finding was comparable with previous reports, including those from Indonesia, showing that the 30-day incidence among HCWs during the first and second year of the COVID-19 pandemic was ranging around 0.5–4%.^19^, ^20^, ^21^, ^22^, ^23^, ^24^ These trends of COVID-19 cases in our cohort corroborated with the national (Indonesia) and regional (East Java) COVID-19 cases which also showed two peaks of COVID-19 at similar times during this study period.^17^^,^^18^

We found that during the first wave of the pandemic, the incidence of COVID-19 among medical staffs (physicians, residents, and medical students) and nurses were higher than in non-medical workers such as pharmacists, technicians and administrative staffs. This is obvious because nurses and medical staffs have more contact with infected patients. Furthermore, previous observations have indicated that HCWs with more direct contact with patients are at higher risk to be infected by the SARS-CoV-2 virus.^20^^,^^21^^,^^23^^,^^24^ Interestingly, during the post-vaccination period the incidence of COVID-19 in medical staff were comparable to incidence in non-medical staffs. The medical staffs, those who worked in the frontline, were prioritised to receive vaccinations. This might explain the finding that in non-medical staff, there was steeper increase in SARS-CoV-2 infection compared to medical staff who received vaccinations. In addition, it is possible that at the later stage of the pandemic, many HCWs had less compliance in using personal protective equipment (PPE). However, it is important to note that there was no change in the official guidelines for HCWs on the use of PPEs during work in hospitals between the pre-vaccination and post-vaccination periods. Thus, any reduction in compliance might be related to the decrease in anxiety level and less fear of contracting COVID-19 in HCWs following complete vaccination, as indicated by a study in South Korea.^25^ Indeed, risk perception and distress are likely to influence the compliance in using correct PPE as suggested in previous observations.^26^^,^^27^

Our study provides a remarkable finding that the cumulative incidence of COVID-19 was higher during the post-vaccination period compared to that of the pre-vaccination period. Detailed analysis of the data revealed that the incidence of COVID-19 declined during the first three months of the vaccination programme. However, by the end of May 2021 the infection rate was dramatically increased, which coincided with the emergence of the Delta variant of SARS-CoV-2 virus. The increase in SARS-CoV-2 infection during this period is in line with the regional (East Java) and national (Indonesian) epidemic curve.^17^ It is very likely that the protective effect of the inactivated viral vaccine was significantly reduced against the Delta variant as indicated by the marked decline in vaccine effectiveness from 73.3% to 17.6% when Delta variant became the dominant variant in the province. This agrees with previous observations showing the reduction of vaccine effectiveness against the emerging Delta variant.^28^, ^29^, ^30^, ^31^ However, it is also important to note that although the infection rate was higher in the post-vaccination period, the COVID-19 incidence during this time was significantly lower in fully vaccinated participants compared to incomplete/unvaccinated individuals (21.4% vs 38.1%, Table 3). This finding suggests that there is still some degree of protection of vaccination despite the emergence of the Delta variant.

Another possible explanation is the waning of the immune protection overtime. Our separate study revealed that the serum antibody levels in HCWs vaccinated with inactivated viral vaccine was at the highest at one month following vaccination and then gradually decreased at 3–5 months after vaccination.^32^ This finding is in line with similar observations using different types of COVID-19 vaccines.^7^^,^^8^^,^^33^ As the peak of COVID-19 infection in our cohort occurred around 3–5 months after the vaccination programme, it is possible that the reduction of the immune protection in combination with the emergence of Delta variant contributed to the surge of COVID-19 incidence between June–August 2021.

Another important observation in this study is the pattern of hospitalisation in the early phase of the pandemic compared to the post-vaccination period. It is not surprising that the total number of the hospitalised participants was higher in the second period of the study because the total number of infections was significantly higher in that period. However, the proportion of hospitalised subjects/total infected participants was markedly reduced from 23.5% in the pre-vaccination period to 14.3% in the post-vaccination period. This might be due to the combination of several factors. First, the severity of COVID-19 in most of the participants might be milder due to the protective effect of the vaccine. It is widely believed that the vaccination programme has successfully reduced hospitalisation as indicated in some real-world vaccine effectiveness reports^34^, ^35^, ^36^ and this might also be the case in our cohort. Second, the reduction in the rate of hospitalisations might be due to the change in the policy in determining the criteria for hospitalisation. During the second wave of COVID-19 in Indonesia, the Ministry of Health provided guidance with stricter criteria for hospitalisation,^37^^,^^38^ in which only severe patients with hypoxia or those with severe comorbidities were admitted to the hospital. Patients who did not experience hypoxia or breathing difficulties were advised to self-isolate and be treated at their home or at a community quarantine facility. In contrast, in the early phase of the pandemic, the Indonesian authority was more focused on tracing efforts, identifying patients, and preventing transmission in the community, so that all the cases that met the definition criteria of COVID-19 (including those with only mild to moderate symptoms) had to be treated at several referral hospitals determined by the government in each province, district and city for treatment and also for isolation purposes. The change in policy might explain the reduction in the hospitalisation rate and underscore the importance of having a clear protocol from the national authority in providing the best strategy for tackling the surge of COVID-19 cases.

One of the most interesting findings of this study is the analysis of the association between demographic characteristics, the presence of co-morbidities and the likelihood of being hospitalised. Unsurprisingly, males and older subjects (≥50 years) showed higher odds ratio to be hospitalised which agrees with previous reports.^14^^,^^39^^,^^40^ The adjusted odds ratios (aORs) from the multivariate analysis showed that participants with history of hypertension, diabetes mellitus and lung disease as well as BMI≥30 (obesity) also displayed higher chances to be hospitalised. Hypertension appeared to be the strongest determinants in the pre-vaccination period with *P* value of 0.005. Interestingly, a marked reduction in the aORs between the pre- and post-vaccination periods was observed in subjects with hypertension. Of note, there were trends of significance (with *P* value between 0.05 and 0.1) of the aORs of being hospitalized in participants with history of diabetes mellitus, lung diseases and in older participants (age ≥50 years). However, unlike hypertension, the aORs of these comorbidities were comparable between the pre- and post-vaccination periods. Age, sex, and the presence of comorbidities such as hypertension, diabetes mellitus and lung diseases have been strongly associated with the severity of COVID-19 as indicated in previous publications.^14^, ^15^, ^16^ Alteration of the immune system has been proposed as the key mechanism leading to severe COVID-19 in older people^41^ and individuals with comorbidities such as hypertension^42^ and diabetes mellitus.^43^ However, it remains unclear as to why participants with hypertension had reduced risk of being hospitalised after the vaccination programme while participants with other co-morbidities did not display such risk reduction. Further studies are needed to understand this phenomenon. Nevertheless, this finding underlines the importance of protecting vulnerable people with comorbidities by using vaccinations.

Despite the findings, we acknowledge some limitations of the study. First, our study was a retrospective study and some of the data were obtained from the completion of the questionnaire. We acknowledge that there might be some degree of bias in providing information regarding demographic characteristics and the presence of comorbidities. However, since most of the participants were HCWs, we believe that they had a good knowledge concerning health condition and so the possible inaccuracy of the information regarding their health condition should be minimum. Second, we were not able to assess COVID-19 death rates during the period of the study because this study was a retrospective study involving healthcare workers who were non-infected or survived from COVID-19 during the period of the study. Another limitation was regarding the analysis of the dominant SARS-CoV-2 variant during the period of the study. Ideally, the data of SARS-CoV-2 variants should be obtained directly from the participants of this study. However, due to the limitation of the DNA sequencing facilities in our centre, we could only analyse sequence data in the GISAID database. However, we believe that the data accurately represent the dominant strains that circulated in East Java in the respective times.

In conclusion, our analyses provide accurate information on the incidence, hospitalisation, and characteristics of COVID-19 cases among HCWs in East Java, Indonesia comparing between the pre- and post-vaccination periods. We observed that the risk to contract COVID-19 amongst HCWs in two major hospitals in East Java remains high despite the high coverage of vaccination. However, the rate of hospitalisation was reduced in the post-vaccination period. Information concerning the burden due to SARS-CoV-2 infection among HCWs is essential, first to ensure the health and safety of the HCWs, and second to provide representative data on the outbreak itself since the findings may represent what is happening in the wider population. The findings can be used to increase public awareness and to provide the authorities with recommendations related to COVID-19 management, the use PPE among HCWs, and vaccination including the need of booster doses.

## Contributors

GS conceived the original idea, designed the study, collected and analysed all research data, wrote and edited manuscript; DP designed the study, supervised data analysis, edited manuscript; LW designed the study, managed funding, collected and analysed research data; BAM collected clinical data, performed additional data analysis; KDF, STH, HIG, MEP, DP, PPN, NA collected participants' data; CRSP supervised, directed, and coordinated the project; AE supervised the project; DGAS supervised the vaccination programme and the project; DT supervised the ethical clearance; EBR supervised the vaccination programme and the project; EAT supervised the project; JW authorised the vaccination programme at Dr. Soetomo Hospital and supervised the project; CBK, FEW, FM collected participants’ and clinical data at Bangkalan Hospital; NK authorised the vaccination programme at Bangkalan Hospital and supervised the project; AB, DF supervised the project and provided suggestions during manuscript writing; WKN supervised the project; DO designed the study, performed data analysis, and wrote and edited the manuscript. All authors have read and approved the final manuscript.

## Data sharing statement

The datasets created and analysed during this study are available from the corresponding authors upon reasonable request.

## Declaration of interests

None.
