# Supplementary material for: Incidence of SARS-CoV-2 infection in hospital workers before and after vaccination programme in East Java, Indonesia – a retrospective cohort study
Source: Lancet Reg Health Southeast Asia. 2022 Dec 12;10:100130. doi: 10.1016/j.lansea.2022.100130 (PMC9742226; doi:10.1016/j.lansea.2022.100130)
Supplement: Supplementary materials [file mmc1.pdf]

SUPPLEMENTARY MATERIALS

Soegiarto et al. "Incidence of SARS-CoV-2 infection in hospital workers before and after vaccination programme in East Java Indonesia - A retrospective cohort study"

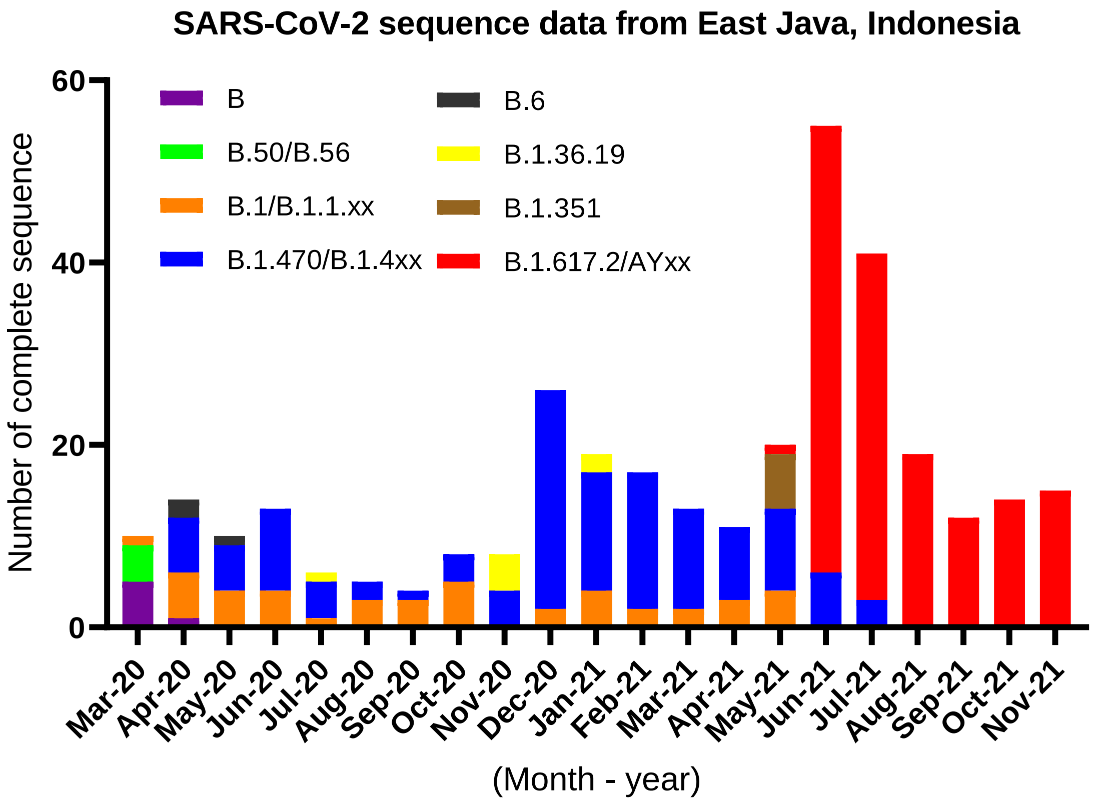

Supplementary Figure 1. The change in the dominant SARS-CoV-2 variant in East Java during the period of the study.

We evaluated all SARS-CoV-2 sequence data originated from East Java, Indonesia between March 2020 – November 2021 that were submitted in GISAID database. The dominant virus variant in East Java during the first wave of pandemic was the Pango lineage B.1.470. It shifted to the variant 1.617.2, also known as Delta variant, during the second surge of infection (Jun 2021 – Nov 2021).

Supplementary Table 1 List of virus sequences from the GISAID database used as the source of analysis presented in Figure 2

Virus name	Accession ID	Collection date	Location	Lineage	Clade
hCoV-19/Indonesia/JI-ITD-136N/2020	EPI_ISL_529961	12/03/2020	Asia / Indonesia / East Java / Surabaya	B	L
hCoV-19/Indonesia/JI-ITD-150Sp/2020	EPI_ISL_529962	16/03/2020	Asia / Indonesia / East Java / Surabaya	B	L
hCoV-19/Indonesia/JI-ITD-853Sp/2020	EPI_ISL_437187	25/03/2020	Asia / Indonesia / East Java / Surabaya	B.50	L
hCoV-19/Indonesia/JI-ITDua-966NTv/2020	EPI_ISL_759966	26/03/2020	Asia / Indonesia / East Java / Sidoarjo	B	L
hCoV-19/Indonesia/JI-ITD-1038NTv/2020	EPI_ISL_2284873	27/03/2020	Asia / Indonesia / East Java / Surabaya	B.1	G
hCoV-19/Indonesia/JI-ITDua-1006NTv/2020	EPI_ISL_759961	27/03/2020	Asia / Indonesia / East Java / Surabaya	B.56	L
hCoV-19/Indonesia/JI-ITD-1238Sp/2020	EPI_ISL_458079	30/03/2020	Asia / Indonesia / East Java / Surabaya	B.56	L
hCoV-19/Indonesia/JI-ITD-1273NT/2020	EPI_ISL_458081	30/03/2020	Asia / Indonesia / East Java / Pasuruan	B	L
hCoV-19/Indonesia/JI-ITD-1273V/2020	EPI_ISL_529964	30/03/2020	Asia / Indonesia / East Java / Pasuruan	B	L
hCoV-19/Indonesia/JI-ITDua-998NTv/2020	EPI_ISL_759959	30/03/2020	Asia / Indonesia / East Java / Surabaya	B.56	L
hCoV-19/Indonesia/JI-ITDua-1609Nv/2020	EPI_ISL_759967	01/04/2020	Asia / Indonesia / East Java / Tulungagung	B.1	GH
hCoV-19/Indonesia/JI-ITD-2766NT/2020	EPI_ISL_458082	09/04/2020	Asia / Indonesia / East Java / Surabaya	B.1	GH
hCoV-19/Indonesia/JI-ITDua-2858NTv/2020	EPI_ISL_759965	09/04/2020	Asia / Indonesia / East Java / Surabaya	B.1.470	GH
hCoV-19/Indonesia/JI-ITD-3101NT/2020	EPI_ISL_458083	11/04/2020	Asia / Indonesia / East Java / Surabaya	B	L
hCoV-19/Indonesia/JI-ITD-3590NT/2020	EPI_ISL_437188	14/04/2020	Asia / Indonesia / East Java / Surabaya	B.1.470	GH
hCoV-19/Indonesia/JI-ITD-3601NT/2020	EPI_ISL_560991	14/04/2020	Asia / Indonesia / East Java / Surabaya	B.6	O
hCoV-19/Indonesia/JI-ITDua-4134NTv/2020	EPI_ISL_759962	17/04/2020	Asia / Indonesia / East Java / Surabaya	B.1.470	GH
hCoV-19/Indonesia/JI-ITDua-4437NTv/2020	EPI_ISL_759955	19/04/2020	Asia / Indonesia / East Java / Surabaya	B.1	GH
hCoV-19/Indonesia/JI-ITD-4859V/2020	EPI_ISL_529965	22/04/2020	Asia / Indonesia / East Java / Surabaya	B.1.470	GH
hCoV-19/Indonesia/JI-ITDua-5235NTv/2020	EPI_ISL_759956	25/04/2020	Asia / Indonesia / East Java / Surabaya	B.1	GH
hCoV-19/Indonesia/JI-ITDua-5392NTv/2020	EPI_ISL_759957	27/04/2020	Asia / Indonesia / East Java / Sidoarjo	B.1.470	GH
hCoV-19/Indonesia/JI-ITDua-5748NTv/2020	EPI_ISL_759958	29/04/2020	Asia / Indonesia / East Java / Surabaya	B.1.470	GH
hCoV-19/Indonesia/JI-ITDua-6033NTv/2020	EPI_ISL_759964	30/04/2020	Asia / Indonesia / East Java / Sidoarjo	B.1	GH
hCoV-19/Indonesia/JI-GSI-P3SCSBY/2020	EPI_ISL_872897	04/05/2020	Asia / Indonesia / East Java	None	O
hCoV-19/Indonesia/JI-ITDua-6647NTv/2020	EPI_ISL_956307	04/05/2020	Asia / Indonesia / East Java / Surabaya	B.1.1	GR
hCoV-19/Indonesia/JI-ITD-7061V/2020	EPI_ISL_529966	05/05/2020	Asia / Indonesia / East Java / Sidoarjo	B.1	GH
hCoV-19/Indonesia/JI-ITD-8402NT/2020	EPI_ISL_529138	11/05/2020	Asia / Indonesia / East Java / Surabaya	B.1	GH
hCoV-19/Indonesia/JI-ITDua-8967NTv/2020	EPI_ISL_956270	12/05/2020	Asia / Indonesia / East Java / Surabaya	B.1.470	GH
hCoV-19/Indonesia/JI-ITDua-9627NTv/2020	EPI_ISL_956308	14/05/2020	Asia / Indonesia / East Java / Surabaya	B.1	GH

hCoV-19/Indonesia/JI-ITDua-11000NTv/2020	EPI_ISL_956271	18/05/2020	Asia / Indonesia / East Java / Surabaya	B.1.470	GH
hCoV-19/Indonesia/JI-RSDS-RCVTD-UNAIR-6B/2020	EPI_ISL_1366083	19/05/2020	Asia / Indonesia / East Java	B.1.470	GH
hCoV-19/Indonesia/JI-RSDS-RCVTD-UNAIR-11-B/2020	EPI_ISL_1366238	19/05/2020	Asia / Indonesia / East Java / Surabaya	B.1.470	G
hCoV-19/Indonesia/JI-ITDua-12009NTv/2020	EPI_ISL_956273	23/05/2020	Asia / Indonesia / East Java / Surabaya	B.1.470	GH
hCoV-19/Indonesia/JI-ITD-12202V/2020	EPI_ISL_529967	09/06/2020	Asia / Indonesia / East Java / Sidoarjo	B.1.470	GH
hCoV-19/Indonesia/JI-ITDua-12323Nvv/2020	EPI_ISL_759963	09/06/2020	Asia / Indonesia / East Java / Sidoarjo	B.1.470	GH
hCoV-19/Indonesia/JI-ITDua-12418Nv/2020	EPI_ISL_956309	10/06/2020	Asia / Indonesia / East Java / Sidoarjo	B.1.470	GH
hCoV-19/Indonesia/JI-NIHRD-PME0999/2020	EPI_ISL_538498	11/06/2020	Asia / Indonesia / East Java	B.1.470	GH
hCoV-19/Indonesia/JI-ITDua-12663Nv/2020	EPI_ISL_956274	13/06/2020	Asia / Indonesia / East Java / Sidoarjo	B.1.456	GH
hCoV-19/Indonesia/JI-RSDS-RCVTD-UNAIR-35-A/2020	EPI_ISL_1364466	24/06/2020	Asia / Indonesia / East Java	B.1	GH
hCoV-19/Indonesia/JI-RSDS-RCVTD-UNAIR-35-B/2020	EPI_ISL_1364467	24/06/2020	Asia / Indonesia / East Java	B.1	GH
hCoV-19/Indonesia/JI-RSDS-RCVTD-UNAIR-35-C/2020	EPI_ISL_1364468	24/06/2020	Asia / Indonesia / East Java	B.1.470	GH
hCoV-19/Indonesia/RSDS-RCVTD-UNAIR-35-C/2020	EPI_ISL_1366269	24/06/2020	Asia / Indonesia / East Java / Surabaya	B.1.470	GH
hCoV-19/Indonesia/JI-RSDS-RCVTD-UNAIR-33-B/2020	EPI_ISL_1366271	24/06/2020	Asia / Indonesia / East Java / Surabaya	B.1	GH
hCoV-19/Indonesia/JI-RSDS-RCVTD-UNAIR-33-C/2020	EPI_ISL_1366273	24/06/2020	Asia / Indonesia / East Java / Surabaya	B.1	GH
hCoV-19/Indonesia/JI-ITDua-13686NTv/2020	EPI_ISL_956276	24/06/2020	Asia / Indonesia / East Java / Sidoarjo	B.1.470	GH
hCoV-19/Indonesia/JI-NIHRD-PME2054/2020	EPI_ISL_538499	29/06/2020	Asia / Indonesia / East Java	B.1.470	GH
hCoV-19/Indonesia/JI-ITDua-16792Nv/2020	EPI_ISL_759960	14/07/2020	Asia / Indonesia / East Java / Surabaya	B.1	GH
hCoV-19/Indonesia/JI-ITDua-16761NTv/2020	EPI_ISL_956310	14/07/2020	Asia / Indonesia / East Java / Surabaya	B.1.470	GH
hCoV-19/Indonesia/JI-ITDua-17047NTv/2020	EPI_ISL_956311	16/07/2020	Asia / Indonesia / East Java / Surabaya	B.1.36.19	GH
hCoV-19/Indonesia/JI-ITD-17398NT/2020	EPI_ISL_529963	18/07/2020	Asia / Indonesia / East Java / Surabaya	B.1.470	GH
hCoV-19/Indonesia/JI-ITDua-17563Nv/2020	EPI_ISL_956277	20/07/2020	Asia / Indonesia / East Java / Surabaya	B.1.470	GH

hCoV-19/Indonesia/JI-ITDua-18214NTv/2020	EPI_ISL_956278	24/07/2020	Asia / Indonesia / East Java / Sidoarjo	B.1.470	GH
hCoV-19/Indonesia/JI-NIHRD-PME5011/2020	EPI_ISL_538511	01/08/2020	Asia / Indonesia / East Java	B.1.456	GH
hCoV-19/Indonesia/JI-ITD-19903NTv/2020	EPI_ISL_1165067	03/08/2020	Asia / Indonesia / East Java / Sidoarjo	B.1.470	GH
hCoV-19/Indonesia/JI-ITD-19882Nv/2020	EPI_ISL_1165075	03/08/2020	Asia / Indonesia / East Java / Surabaya	B.1	GH
hCoV-19/Indonesia/JI-ITD-21907NTv/2020	EPI_ISL_1165068	13/08/2020	Asia / Indonesia / East Java / Sidoarjo	B.1	GH
hCoV-19/Indonesia/JI-ITD-24254NTv/2020	EPI_ISL_1165069	28/08/2020	Asia / Indonesia / East Java / Sidoarjo	B.1	GH
hCoV-19/Indonesia/JI-ITD-25302Nv/2020	EPI_ISL_1159381	04/09/2020	Asia / Indonesia / East Java / Surabaya	B.1	GH
hCoV-19/Indonesia/JI-ITD-25612Nv/2020	EPI_ISL_1165076	05/09/2020	Asia / Indonesia / East Java / Sidoarjo	B.1	GH
hCoV-19/Indonesia/JI-ITD-26463NTv/2020	EPI_ISL_1165070	12/09/2020	Asia / Indonesia / East Java / Sidoarjo	B.1.1.28	GR
hCoV-19/Indonesia/JI-ITD-26529NTv/2020	EPI_ISL_1165071	14/09/2020	Asia / Indonesia / East Java / Sidoarjo	B.1.470	GH
hCoV-19/Indonesia/JI-ITDua-28257NT/2020	EPI_ISL_759968	02/10/2020	Asia / Indonesia / East Java / Surabaya	B.1.470	GH
hCoV-19/Indonesia/JI-NIHRD-PME10137/2020	EPI_ISL_791989	03/10/2020	Asia / Indonesia / East Java	B.1.1	GR
hCoV-19/Indonesia/JI-ITD-28533NTv/2020	EPI_ISL_1789113	06/10/2020	Asia / Indonesia / East Java / Sidoarjo	B.1	GH
hCoV-19/Indonesia/JI-ITD-28656Nv/2020	EPI_ISL_1159382	07/10/2020	Asia / Indonesia / East Java / Surabaya	B.1.470	GH
hCoV-19/Indonesia/JI-ITD-28759NTv/2020	EPI_ISL_1789114	09/10/2020	Asia / Indonesia / East Java / Sidoarjo	B.1.470	GH
hCoV-19/Indonesia/JI-ITD-29089NTv/2020	EPI_ISL_1159383	14/10/2020	Asia / Indonesia / East Java / Sidoarjo	B.1	GH
hCoV-19/Indonesia/JI-ITD-29375NTv/2020	EPI_ISL_1159384	19/10/2020	Asia / Indonesia / East Java / Surabaya	B.1.1.398	GR
hCoV-19/Indonesia/JI-ITD-29881NTv/2020	EPI_ISL_1159385	26/10/2020	Asia / Indonesia / East Java / Surabaya	B.1	GH
hCoV-19/Indonesia/JI-ITD-30338NTv/2020	EPI_ISL_1159386	03/11/2020	Asia / Indonesia / East Java / Sidoarjo	B.1.36.19	GH
hCoV-19/Indonesia/JI-ITD-30604Nv/2020	EPI_ISL_1159387	09/11/2020	Asia / Indonesia / East Java / Surabaya	B.1.470	GH
hCoV-19/Indonesia/JI-NIHRD-PME9750/2020	EPI_ISL_791980	09/11/2020	Asia / Indonesia / East Java	B.1.456	GH
hCoV-19/Indonesia/JI-ITD-30833Nv/2020	EPI_ISL_1159388	13/11/2020	Asia / Indonesia / East Java / Surabaya	B.1.470	GH
hCoV-19/Indonesia/JI-ITD-31235NTv/2020	EPI_ISL_1159389	20/11/2020	Asia / Indonesia / East Java / Surabaya	B.1.36.19	GH
hCoV-19/Indonesia/JI-ITD-31548NTv/2020	EPI_ISL_1789115	24/11/2020	Asia / Indonesia / East Java / Sidoarjo	B.1.36.19	GH
hCoV-19/Indonesia/JI-ITD-32011NTv/2020	EPI_ISL_2274436	30/11/2020	Asia / Indonesia / East Java / Sidoarjo	B.1.36.19	GH
hCoV-19/Indonesia/JI-ITD-32072NTv/2020	EPI_ISL_2284874	30/11/2020	Asia / Indonesia / East Java / Sidoarjo	B.1.456	GH
hCoV-19/Indonesia/JI-ITD-32113NTv/2020	EPI_ISL_1789116	01/12/2020	Asia / Indonesia / East Java / Sidoarjo	B.1.1.398	GR
hCoV-19/Indonesia/JI-ITD-32118NTv/2020	EPI_ISL_1795053	01/12/2020	Asia / Indonesia / East Java / Sidoarjo	B.1.470	GH
hCoV-19/Indonesia/JI-ITD-32173NTv/2020	EPI_ISL_1795054	01/12/2020	Asia / Indonesia / East Java / Surabaya	B.1.470	GH

hCoV-19/Indonesia/JI-ITD-32681NTv/2020	EPI_ISL_1795055	07/12/2020	Asia / Indonesia / East Java / Gresik	B.1.470	GH
hCoV-19/Indonesia/JI-ITD-33091Nv/2020	EPI_ISL_1795056	08/12/2020	Asia / Indonesia / East Java / Surabaya	B.1.456	GH
hCoV-19/Indonesia/JI-ITD-33120NTv/2020	EPI_ISL_1795057	09/12/2020	Asia / Indonesia / East Java / Gresik	B.1.470	GH
hCoV-19/Indonesia/JI-ITDua-33281NT/2020	EPI_ISL_877419	11/12/2020	Asia / Indonesia / East Java / Sidoarjo	B.1.466.2	GH
hCoV-19/Indonesia/JI-ITDua-33304NT/2020	EPI_ISL_877420	11/12/2020	Asia / Indonesia / East Java / Surabaya	B.1.470	GH
hCoV-19/Indonesia/JI-ITDua-33312NT/2020	EPI_ISL_877421	11/12/2020	Asia / Indonesia / East Java / Surabaya	B.1.470	GH
hCoV-19/Indonesia/JI-ITDua-33753N/2020	EPI_ISL_877422	15/12/2020	Asia / Indonesia / East Java / Sidoarjo	B.1.470	GH
hCoV-19/Indonesia/JI-ITDua-33816NT/2020	EPI_ISL_877423	16/12/2020	Asia / Indonesia / East Java / Surabaya	B.1.470	GH
hCoV-19/Indonesia/JI-ITDua-33807NT/2020	EPI_ISL_877456	16/12/2020	Asia / Indonesia / East Java / Surabaya	B.1.466.2	GH
hCoV-19/Indonesia/JI-ITDua-33817NT/2020	EPI_ISL_877457	16/12/2020	Asia / Indonesia / East Java / Surabaya	B.1.459	GH
hCoV-19/Indonesia/JI-ITDua-33862NT/2020	EPI_ISL_877458	16/12/2020	Asia / Indonesia / East Java / Sidoarjo	B.1.468	GH
hCoV-19/Indonesia/JI-ITDua-34192NT/2020	EPI_ISL_877459	18/12/2020	Asia / Indonesia / East Java / Surabaya	B.1.470	GH
hCoV-19/Indonesia/JI-ITDua-34158NT/2020	EPI_ISL_882769	18/12/2020	Asia / Indonesia / East Java / Surabaya	B.1.456	GH
hCoV-19/Indonesia/JI-ITDua-34660NT/2020	EPI_ISL_877460	22/12/2020	Asia / Indonesia / East Java / Sidoarjo	B.1.459	GH
hCoV-19/Indonesia/JI-ITDua-34618NT/2020	EPI_ISL_882770	22/12/2020	Asia / Indonesia / East Java / Surabaya	B.1.470	GH
hCoV-19/Indonesia/JI-ITD-34777NTv/2020	EPI_ISL_1795058	23/12/2020	Asia / Indonesia / East Java / Surabaya	B.1.470	GH
hCoV-19/Indonesia/JI-ITDua-34962NT/2020	EPI_ISL_888591	24/12/2020	Asia / Indonesia / East Java / Sidoarjo	B.1.470	GH
hCoV-19/Indonesia/JI-ITDua-34965NT/2020	EPI_ISL_888593	24/12/2020	Asia / Indonesia / East Java / Sidoarjo	B.1.470	GH
hCoV-19/Indonesia/JI-ITDua-35379NT/2020	EPI_ISL_877538	28/12/2020	Asia / Indonesia / East Java / Sidoarjo	B.1.470	GH
hCoV-19/Indonesia/JI-ITDua-35340NT/2020	EPI_ISL_882771	28/12/2020	Asia / Indonesia / East Java / Sidoarjo	B.1.470	GH
hCoV-19/Indonesia/JI-ITD-35608Nv/2020	EPI_ISL_1795059	29/12/2020	Asia / Indonesia / East Java / Sidoarjo	B.1.470	GH
hCoV-19/Indonesia/JI-ITDua-35767NT/2020	EPI_ISL_956279	30/12/2020	Asia / Indonesia / East Java / Sidoarjo	B.1.470	GH
hCoV-19/Indonesia/JI-ITD-35800NTv/2020	EPI_ISL_1795060	31/12/2020	Asia / Indonesia / East Java / Surabaya	B.1.110	GH
hCoV-19/Indonesia/JI-ITDua-35937NT/2021	EPI_ISL_956312	01/01/2021	Asia / Indonesia / East Java / Surabaya	B.1.470	GH
hCoV-19/Indonesia/JI-ITD-36311NTv/2021	EPI_ISL_1789117	04/01/2021	Asia / Indonesia / East Java / Sidoarjo	B.1.470	GH
hCoV-19/Indonesia/JI-ITDua-36208NT/2021	EPI_ISL_956280	04/01/2021	Asia / Indonesia / East Java / Surabaya	B.1.470	GH
hCoV-19/Indonesia/JI-ITDua-36471NT/2021	EPI_ISL_956281	04/01/2021	Asia / Indonesia / East Java / Sidoarjo	B.1.470	GH
hCoV-19/Indonesia/JI-ITDua-36484NT/2021	EPI_ISL_956315	04/01/2021	Asia / Indonesia / East Java / Sidoarjo	B.1.470	GH
hCoV-19/Indonesia/JI-ITDua-36613NT/2021	EPI_ISL_956316	06/01/2021	Asia / Indonesia / East Java / Surabaya	B.1.470	GH
hCoV-19/Indonesia/JI-ITDua-36824NT/2021	EPI_ISL_956313	08/01/2021	Asia / Indonesia / East Java / Sidoarjo	B.1.36.19	GH
hCoV-19/Indonesia/JI-ITDua-37008N/2021	EPI_ISL_956314	11/01/2021	Asia / Indonesia / East Java / Surabaya	B.1.470	GH

hCoV-19/Indonesia/JI-RSDS-RCVTD-UNAIR-54-A/2021	EPI_ISL_1366503	12/01/2021	Asia / Indonesia / East Java / Surabaya	B.1	GH
hCoV-19/Indonesia/JI-ITD-37255NT/2021	EPI_ISL_1165072	14/01/2021	Asia / Indonesia / East Java / Surabaya	B.1.466.2	GH
hCoV-19/Indonesia/JI-ITD-37338NT/2021	EPI_ISL_1165073	14/01/2021	Asia / Indonesia / East Java / Surabaya	B.1.456	GH
hCoV-19/Indonesia/JI-ITD-37257NTv/2021	EPI_ISL_1789118	14/01/2021	Asia / Indonesia / East Java / Surabaya	B.1.470	GH
hCoV-19/Indonesia/JI-ITD-37619NTv/2021	EPI_ISL_1789119	18/01/2021	Asia / Indonesia / East Java / Surabaya	B.1.466.2	GH
hCoV-19/Indonesia/JI-RSDS-RCVTD-UNAIR-49-A/2021	EPI_ISL_1366505	21/01/2021	Asia / Indonesia / East Java / Surabaya	B.1	GH
hCoV-19/Indonesia/JI-RSDS-RCVTD-UNAIR-42-A/2021	EPI_ISL_1366509	21/01/2021	Asia / Indonesia / East Java / Surabaya	B.1	GH
hCoV-19/Indonesia/JI-ITD-38076NTv/2021	EPI_ISL_1789172	22/01/2021	Asia / Indonesia / East Java / Surabaya	B.1.470	GH
hCoV-19/Indonesia/JI-ITD-38279NT/2021	EPI_ISL_1159390	25/01/2021	Asia / Indonesia / East Java / Gresik	B.1	GH
hCoV-19/Indonesia/JI-ITD-38281N/2021	EPI_ISL_1789112	25/01/2021	Asia / Indonesia / East Java / Pasuruan	B.1.470	GH
hCoV-19/Indonesia/JI-ITD-38310Nv/2021	EPI_ISL_1789202	25/01/2021	Asia / Indonesia / East Java / Surabaya	B.1.36.19	GH
hCoV-19/Indonesia/JI-ITD-38912NT/2021	EPI_ISL_1165074	02/02/2021	Asia / Indonesia / East Java / Surabaya	B.1.470	GH
hCoV-19/Indonesia/JI-ITD-39066NTv/2021	EPI_ISL_2284875	03/02/2021	Asia / Indonesia / East Java / Surabaya	B.1.470	GH
hCoV-19/Indonesia/JI-NIHRD-WGS01173/2021	EPI_ISL_1469246	04/02/2021	Asia / Indonesia / East Java	B.1.470	GH
hCoV-19/Indonesia/JI-NIHRD-WGS01175/2021	EPI_ISL_1469271	04/02/2021	Asia / Indonesia / East Java	B.1.470	GH
hCoV-19/Indonesia/JI-NIHRD-WGS01179/2021	EPI_ISL_1469272	08/02/2021	Asia / Indonesia / East Java	B.1.466.2	GH
hCoV-19/Indonesia/JI-ITD-39254NTv/2021	EPI_ISL_2284876	08/02/2021	Asia / Indonesia / East Java / Surabaya	B.1	GH
hCoV-19/Indonesia/JI-ITD-39376NT/2021	EPI_ISL_1159391	09/02/2021	Asia / Indonesia / East Java / Surabaya	B.1.470	GH
hCoV-19/Indonesia/JI-PNF-211352/2021	EPI_ISL_6425650	10/02/2021	Asia / Indonesia / East Java / Surabaya	B.1.470	GH
hCoV-19/Indonesia/JI-PNF-211373/2021	EPI_ISL_6425649	11/02/2021	Asia / Indonesia / East Java / Sidoarjo	B.1.470	GH
hCoV-19/Indonesia/JI-ITD-39660NTv/2021	EPI_ISL_2284877	15/02/2021	Asia / Indonesia / East Java / Surabaya	B.1.466.2	GH
hCoV-19/Indonesia/JI-NIHRD-WGS01181/2021	EPI_ISL_1469273	16/02/2021	Asia / Indonesia / East Java	B.1.459	GH
hCoV-19/Indonesia/JI-NIHRD-WGS01183/2021	EPI_ISL_1469274	16/02/2021	Asia / Indonesia / East Java	B.1.1.398	GR

hCoV-19/Indonesia/JI-NIHRD-WGS01184/2021	EPI_ISL_1469275	16/02/2021	Asia / Indonesia / East Java	B.1.466.2	GH
hCoV-19/Indonesia/JI-NIHRD-WGS01186/2021	EPI_ISL_1469240	18/02/2021	Asia / Indonesia / East Java	B.1	GH
hCoV-19/Indonesia/JI-NIHRD-WGS01185/2021	EPI_ISL_1469284	18/02/2021	Asia / Indonesia / East Java	B.1.466.2	GH
hCoV-19/Indonesia/JI-ITD-39869NTv/2021	EPI_ISL_2284878	18/02/2021	Asia / Indonesia / East Java / Surabaya	B.1.470	GH
hCoV-19/Indonesia/JI-ITD-39945NTv/2021	EPI_ISL_2284879	19/02/2021	Asia / Indonesia / East Java / Sidoarjo	B.1.470	GH
hCoV-19/Indonesia/JI-ITD-40187NTv/2021	EPI_ISL_2284880	24/02/2021	Asia / Indonesia / East Java / Surabaya	B.1.470	GH
hCoV-19/Indonesia/JI-ITD-40538NTv/2021	EPI_ISL_2284881	03/03/2021	Asia / Indonesia / East Java / Surabaya	B.1.470	GH
hCoV-19/Indonesia/JI-ITD-40544NTv/2021	EPI_ISL_2284882	03/03/2021	Asia / Indonesia / East Java / Surabaya	B.1.470	GH
hCoV-19/Indonesia/JI-ITD-40618NTv/2021	EPI_ISL_2284883	05/03/2021	Asia / Indonesia / East Java / Sidoarjo	B.1.470	GH
hCoV-19/Indonesia/JI-ITD-43154Nv/2021	EPI_ISL_2284869	09/03/2021	Asia / Indonesia / East Java / Surabaya	B.1	GH
hCoV-19/Indonesia/JI-NIHRD-WGS01832/2021	EPI_ISL_1469250	10/03/2021	Asia / Indonesia / East Java	B.1.470	GH
hCoV-19/Indonesia/JI-ITD-43155Nv/2021	EPI_ISL_2284870	10/03/2021	Asia / Indonesia / East Java / Surabaya	B.1.470	GH
hCoV-19/Indonesia/JI-ITD-43156Nv/2021	EPI_ISL_2284871	14/03/2021	Asia / Indonesia / East Java / Surabaya	B.1	GH
hCoV-19/Indonesia/JI-ITD-43158Nv/2021	EPI_ISL_2274437	15/03/2021	Asia / Indonesia / East Java / Surabaya	B.1.466.2	GH
hCoV-19/Indonesia/JI-ITD-43157Nv/2021	EPI_ISL_2284872	15/03/2021	Asia / Indonesia / East Java / Surabaya	B.1.466.2	GH
hCoV-19/Indonesia/JI-ITD-43159Nv/2021	EPI_ISL_2284884	16/03/2021	Asia / Indonesia / East Java / Surabaya	B.1.470	GH
hCoV-19/Indonesia/JI-ITD-41307NTv/2021	EPI_ISL_2274438	20/03/2021	Asia / Indonesia / East Java / Surabaya	B.1.470	GH
hCoV-19/Indonesia/JI-NIHRD-WGS02633/2021	EPI_ISL_2047570	22/03/2021	Asia / Indonesia / East Java	B.1.470	GH
hCoV-19/Indonesia/JI-ITD-43153Nv/2021	EPI_ISL_2284868	29/03/2021	Asia / Indonesia / East Java / Surabaya	B.1.466.2	GH
hCoV-19/Indonesia/JI-NIHRD-WGS02634/2021	EPI_ISL_2047571	05/04/2021	Asia / Indonesia / East Java	B.1.470	GH
hCoV-19/Indonesia/JI-ITD-42606N/2021	EPI_ISL_2226647	21/04/2021	Asia / Indonesia / East Java / Surabaya	B.1.1.7	GRY
hCoV-19/Indonesia/JI-NIHRD-WGS03205/2021	EPI_ISL_2258207	21/04/2021	Asia / Indonesia / East Java / Surabaya	B.1.1.7	GR
hCoV-19/Indonesia/JI-NIHRD-WGS03204/2021	EPI_ISL_2262277	21/04/2021	Asia / Indonesia / East Java / Surabaya	B.1.466.2	GH

hCoV-19/Indonesia/JI-NIHRD-WGS02910/2021	EPI_ISL_2854695	22/04/2021	Asia / Indonesia / East Java	B.1.466.2	GH
hCoV-19/Indonesia/JI-NIHRD-WGS02911/2021	EPI_ISL_2854696	23/04/2021	Asia / Indonesia / East Java	B.1.1.419	GR
hCoV-19/Indonesia/JI-NIHRD-WGS03202/2021	EPI_ISL_2262276	25/04/2021	Asia / Indonesia / East Java / Surabaya	B.1.466.2	GH
hCoV-19/Indonesia/JI-GS-ITD-42414N/2021	EPI_ISL_7189399	26/04/2021	Asia / Indonesia / East Java / Surabaya	B.1.470	GH
hCoV-19/Indonesia/JI-GS-ITD-42463NT/2021	EPI_ISL_7189408	26/04/2021	Asia / Indonesia / East Java / Surabaya	B.1.466.2	GH
hCoV-19/Indonesia/JI-NIHRD-WGS02909/2021	EPI_ISL_2854694	27/04/2021	Asia / Indonesia / East Java	B.1.466.2	GH
hCoV-19/Indonesia/JI-GS-ITD-42524NT/2021	EPI_ISL_7189417	28/04/2021	Asia / Indonesia / East Java / Surabaya	B.1.470	GH
hCoV-19/Indonesia/JI-NIHRD-WGS03210/2021	EPI_ISL_2854699	03/05/2021	Asia / Indonesia / East Java	B.1.466.2	GH
hCoV-19/Indonesia/JI-NIHRD-WGS03211/2021	EPI_ISL_2854700	03/05/2021	Asia / Indonesia / East Java	B.1.466.2	GH
hCoV-19/Indonesia/JI-ITD-42727N/2021	EPI_ISL_2226648	05/05/2021	Asia / Indonesia / East Java / Surabaya	B.1.351	GH
hCoV-19/Indonesia/JI-NIHRD-c.00.21.34336/2021	EPI_ISL_2617464	05/05/2021	Asia / Indonesia / East Java	B.1.466.2	GH
hCoV-19/Indonesia/JI-NIHRD-c.00.21.34337/2021	EPI_ISL_2617465	05/05/2021	Asia / Indonesia / East Java	B.1.466.2	GH
hCoV-19/Indonesia/JI-NIHRD-WGS03600/2021	EPI_ISL_2868931	05/05/2021	Asia / Indonesia / East Java	None	G
hCoV-19/Indonesia/JI-NIHRD-WGS03630/2021	EPI_ISL_3138809	06/05/2021	Asia / Indonesia / East Java / Kediri	B.1.351	GH
hCoV-19/Indonesia/JI-ITD-42745Nv/2021	EPI_ISL_4056004	07/05/2021	Asia / Indonesia / East Java / Surabaya	B.1.351	GH
hCoV-19/Indonesia/JI-ITD-42829Nv/2021	EPI_ISL_4056005	12/05/2021	Asia / Indonesia / East Java / Surabaya	B.1.351	GH
hCoV-19/Indonesia/JI-ITD-42832Nv/2021	EPI_ISL_4056006	12/05/2021	Asia / Indonesia / East Java / Surabaya	B.1.351	GH
hCoV-19/Indonesia/JI-ITD-42834Nv/2021	EPI_ISL_4056007	12/05/2021	Asia / Indonesia / East Java / Surabaya	None	G
hCoV-19/Indonesia/JI-ITD-42835Nv/2021	EPI_ISL_4056008	12/05/2021	Asia / Indonesia / East Java / Surabaya	B.1.351	GH
hCoV-19/Indonesia/JI-ITD-42831N/2021	EPI_ISL_4742352	12/05/2021	Asia / Indonesia / East Java / Surabaya	B.1.1.7	GR
hCoV-19/Indonesia/JI-NIHRD-WGS05033/2021	EPI_ISL_2987634	26/05/2021	Asia / Indonesia / East Java	AY.23	GK

hCoV-19/Indonesia/JI-GS-ITD-43249N/2021	EPI_ISL_5884798	26/05/2021	Asia / Indonesia / East Java / Surabaya	B.1.1.7	GR
hCoV-19/Indonesia/JI-ITD-43184NTv/2021	EPI_ISL_4056009	27/05/2021	Asia / Indonesia / East Java / Surabaya	B.1.466.2	GH
hCoV-19/Indonesia/JI-ITD-43356NTv/2021	EPI_ISL_3267186	28/05/2021	Asia / Indonesia / East Java / Surabaya	B.1.466.2	GH
hCoV-19/Indonesia/JI-ITD-43254NTv/2021	EPI_ISL_4056010	28/05/2021	Asia / Indonesia / East Java / Malang	B.1.466.2	GH
hCoV-19/Indonesia/JI-ITD-43285Nv/2021	EPI_ISL_3279412	31/05/2021	Asia / Indonesia / East Java / Sampang	B.1.470	GH
hCoV-19/Indonesia/JI-GS-ITD-43285N/2021	EPI_ISL_5884799	31/05/2021	Asia / Indonesia / East Java / Sampang	B.1.470	GH
hCoV-19/Indonesia/JI-ITD-43355NTv/2021	EPI_ISL_4056011	02/06/2021	Asia / Indonesia / East Java / Surabaya	B.1.466.2	GH
hCoV-19/Indonesia/JI-ITD-43358NTv/2021	EPI_ISL_4056012	02/06/2021	Asia / Indonesia / East Java / Surabaya	B.1.466.2	GH
hCoV-19/Indonesia/JI-NIHRD-WGS10118/2021	EPI_ISL_4254872	02/06/2021	Asia / Indonesia / East Java / Malang	AY.24	GK
hCoV-19/Indonesia/JI-NIHRD-WGS10119/2021	EPI_ISL_4254873	02/06/2021	Asia / Indonesia / East Java / Malang	AY.24	GK
hCoV-19/Indonesia/JI-NIHRD-WGS10120/2021	EPI_ISL_4254874	03/06/2021	Asia / Indonesia / East Java / Malang	AY.23	GK
hCoV-19/Indonesia/JI-ITD-43689N/2021	EPI_ISL_2617439	04/06/2021	Asia / Indonesia / East Java / Surabaya	AY.24	GK
hCoV-19/Indonesia/JI-NIHRD-WGS10122/2021	EPI_ISL_4254876	04/06/2021	Asia / Indonesia / East Java / Malang	AY.23	GK
hCoV-19/Indonesia/JI-GS-ITD-43466NT/2021	EPI_ISL_5884800	04/06/2021	Asia / Indonesia / East Java / Surabaya	B.1.470	GH
hCoV-19/Indonesia/JI-NIHRD-WGS10121/2021	EPI_ISL_4254875	05/06/2021	Asia / Indonesia / East Java / Malang	AY.23	GK
hCoV-19/Indonesia/JI-ITD-43589N/2021	EPI_ISL_2510687	06/06/2021	Asia / Indonesia / East Java / Surabaya	AY.24	GK
hCoV-19/Indonesia/JI-ITD-43547N/2021	EPI_ISL_2570805	06/06/2021	Asia / Indonesia / East Java / Surabaya	AY.24	GK
hCoV-19/Indonesia/JI-ITD-43550N/2021	EPI_ISL_2570806	06/06/2021	Asia / Indonesia / East Java / Surabaya	AY.24	GK
hCoV-19/Indonesia/JI-ITD-43574NT/2021	EPI_ISL_2570808	06/06/2021	Asia / Indonesia / East Java / Surabaya	AY.24	GK
hCoV-19/Indonesia/JI-ITD-43584N/2021	EPI_ISL_2570809	06/06/2021	Asia / Indonesia / East Java / Surabaya	AY.24	GK
hCoV-19/Indonesia/JI-ITD-43591N/2021	EPI_ISL_2570811	06/06/2021	Asia / Indonesia / East Java / Surabaya	AY.24	GK
hCoV-19/Indonesia/JI-ITD-43571NT/2021	EPI_ISL_2617435	06/06/2021	Asia / Indonesia / East Java / Surabaya	AY.24	GK
hCoV-19/Indonesia/JI-ITD-43575N/2021	EPI_ISL_2617436	06/06/2021	Asia / Indonesia / East Java / Surabaya	AY.24	GK
hCoV-19/Indonesia/JI-ITD-43585N/2021	EPI_ISL_2617437	06/06/2021	Asia / Indonesia / East Java / Surabaya	AY.24	GK
hCoV-19/Indonesia/JI-ITD-43595NT/2021	EPI_ISL_2617438	06/06/2021	Asia / Indonesia / East Java / Surabaya	B.1.466.2	GH
hCoV-19/Indonesia/JI-ITD-43592N/2021	EPI_ISL_2693669	06/06/2021	Asia / Indonesia / East Java / Surabaya	AY.23	GK
hCoV-19/Indonesia/JI-ITD-43576N/2021	EPI_ISL_2693670	06/06/2021	Asia / Indonesia / East Java / Surabaya	AY.24	GK

hCoV-19/Indonesia/JI-ITD-43688NT/2021	EPI_ISL_2693671	06/06/2021	Asia / Indonesia / East Java / Surabaya	AY.24	GK
hCoV-19/Indonesia/JI-NIHRD-WGS08014/2021	EPI_ISL_3138863	06/06/2021	Asia / Indonesia / East Java / Malang	AY.51	GK
hCoV-19/Indonesia/JI-NIHRD-WGS08016/2021	EPI_ISL_3138865	06/06/2021	Asia / Indonesia / East Java / Malang	AY.23	GK
hCoV-19/Indonesia/JI-NIHRD-WGS10123/2021	EPI_ISL_4254877	06/06/2021	Asia / Indonesia / East Java / Malang	AY.24	GK
hCoV-19/Indonesia/JI-ITD-43568NTv/2021	EPI_ISL_3267187	07/06/2021	Asia / Indonesia / East Java / Lamongan	AY.23	GK
hCoV-19/Indonesia/JI-ITD-43811Nv/2021	EPI_ISL_4056013	11/06/2021	Asia / Indonesia / East Java / Surabaya	AY.24	GK
hCoV-19/Indonesia/JI-ITD-43841NTv/2021	EPI_ISL_4056014	11/06/2021	Asia / Indonesia / East Java / Surabaya	AY.24	GK
hCoV-19/Indonesia/JI-GS-ITD-52548N/2021	EPI_ISL_7189629	12/06/2021	Asia / Indonesia / East Java / Surabaya	AY.24	GK
hCoV-19/Indonesia/JI-GS-ITD-52549N/2021	EPI_ISL_7189638	12/06/2021	Asia / Indonesia / East Java / Surabaya	AY.24	GK
hCoV-19/Indonesia/JI-GS-ITD-52553N/2021	EPI_ISL_7189647	12/06/2021	Asia / Indonesia / East Java / Surabaya	B.1.466.2	GH
hCoV-19/Indonesia/JI-GS-ITD-52577N/2021	EPI_ISL_7189658	12/06/2021	Asia / Indonesia / East Java / Surabaya	AY.23	GK
hCoV-19/Indonesia/JI-GS-ITD-52579N/2021	EPI_ISL_7189666	12/06/2021	Asia / Indonesia / East Java / Surabaya	AY.24	GK
hCoV-19/Indonesia/JI-GS-ITD-52583N/2021	EPI_ISL_7189674	12/06/2021	Asia / Indonesia / East Java / Surabaya	AY.24	GK
hCoV-19/Indonesia/JI-ITD-44415NTv/2021	EPI_ISL_3279414	14/06/2021	Asia / Indonesia / East Java / Malang	AY.23	GK
hCoV-19/Indonesia/JI-GS-ITD-44376NT/2021	EPI_ISL_5884801	14/06/2021	Asia / Indonesia / East Java / Situbondo	AY.24	GK
hCoV-19/Indonesia/JI-GS-ITD-44391NT/2021	EPI_ISL_5884802	14/06/2021	Asia / Indonesia / East Java / Tuban	AY.24	GK
hCoV-19/Indonesia/JI-ITD-43992Nv/2021	EPI_ISL_3279413	15/06/2021	Asia / Indonesia / East Java / Pasuruan	AY.23	GK
hCoV-19/Indonesia/JI-NIHRD-WGS10127/2021	EPI_ISL_4254880	15/06/2021	Asia / Indonesia / East Java / Malang	AY.23	GK
hCoV-19/Indonesia/JI-GS-ITD-44577NT/2021	EPI_ISL_5884804	15/06/2021	Asia / Indonesia / East Java / Kediri	AY.23	GK
hCoV-19/Indonesia/JI-ITD-44255NTv/2021	EPI_ISL_4056015	17/06/2021	Asia / Indonesia / East Java / Mojokerto	AY.24	GK
hCoV-19/Indonesia/JI-GS-ITD-51940N/2021	EPI_ISL_5884833	17/06/2021	Asia / Indonesia / East Java / Probolinggo	B.1.466.2	GH
hCoV-19/Indonesia/JI-GS-ITD-52126NT/2021	EPI_ISL_5884838	17/06/2021	Asia / Indonesia / East Java / Blitar	AY.24	GK
hCoV-19/Indonesia/JI-GS-ITD-52129NT/2021	EPI_ISL_5884839	17/06/2021	Asia / Indonesia / East Java / Blitar	AY.24	GK
hCoV-19/Indonesia/JI-GS-ITD-44571NT/2021	EPI_ISL_5884803	19/06/2021	Asia / Indonesia / East Java / Ponorogo	AY.23	GK
hCoV-19/Indonesia/JI-GS-ITD-51949N/2021	EPI_ISL_5884834	21/06/2021	Asia / Indonesia / East Java / Probolinggo	AY.24	GK
hCoV-19/Indonesia/JI-NIHRD-WGS07799/2021	EPI_ISL_4090553	22/06/2021	Asia / Indonesia / East Java / Magetan	AY.23	GK

hCoV-19/Indonesia/JI-NIHRD-WGS08015/2021	EPI_ISL_3138864	23/06/2021	Asia / Indonesia / East Java / Malang	AY.23	GK
hCoV-19/Indonesia/JI-NIHRD-WGS07798/2021	EPI_ISL_4090552	23/06/2021	Asia / Indonesia / East Java / Magetan	AY.23	GK
hCoV-19/Indonesia/JI-NIHRD-WGS07800/2021	EPI_ISL_4090554	23/06/2021	Asia / Indonesia / East Java / Magetan	AY.23	GK
hCoV-19/Indonesia/JI-GS-ITD-52125NT/2021	EPI_ISL_5884837	23/06/2021	Asia / Indonesia / East Java / Blitar	AY.24	GK
hCoV-19/Indonesia/JI-NIHRD-WGS07801/2021	EPI_ISL_4090555	24/06/2021	Asia / Indonesia / East Java / Magetan	AY.23	GK
hCoV-19/Indonesia/JI-GS-ITD-45299NT/2021	EPI_ISL_7189426	24/06/2021	Asia / Indonesia / East Java / Jombang	AY.24	GK
hCoV-19/Indonesia/JI-PNF-213674/2021	EPI_ISL_6425643	30/06/2021	Asia / Indonesia / East Java / Surabaya	AY.23	GK
hCoV-19/Indonesia/JI-PNF-213673/2021	EPI_ISL_6425645	30/06/2021	Asia / Indonesia / East Java / Surabaya	AY.23	GK
hCoV-19/Indonesia/JI-GS-ITD-46037NT/2021	EPI_ISL_5884805	05/07/2021	Asia / Indonesia / East Java / Surabaya	AY.24	GK
hCoV-19/Indonesia/JI-GS-ITD-46099NT/2021	EPI_ISL_5884806	05/07/2021	Asia / Indonesia / East Java / Gresik	AY.24	GK
hCoV-19/Indonesia/JI-GS-ITD-46107NT/2021	EPI_ISL_5884807	05/07/2021	Asia / Indonesia / East Java / Gresik	AY.23	GK
hCoV-19/Indonesia/JI-GS-ITD-52137NT/2021	EPI_ISL_7189523	07/07/2021	Asia / Indonesia / East Java / Surabaya	AY.24	GK
hCoV-19/Indonesia/JI-GS-ITD-52138NT/2021	EPI_ISL_7189529	07/07/2021	Asia / Indonesia / East Java / Trenggalek	AY.24	GK
hCoV-19/Indonesia/JI-GS-ITD-52539NT/2021	EPI_ISL_7189601	09/07/2021	Asia / Indonesia / East Java / Blitar	AY.23	GK
hCoV-19/Indonesia/JI-GS-ITD-52541NT/2021	EPI_ISL_7189610	09/07/2021	Asia / Indonesia / East Java / Blitar	AY.23	GK
hCoV-19/Indonesia/JI-GS-ITD-52542NT/2021	EPI_ISL_7189622	09/07/2021	Asia / Indonesia / East Java / Blitar	AY.23	GK
hCoV-19/Indonesia/JI-GS-ITD-46736NT/2021	EPI_ISL_5884808	12/07/2021	Asia / Indonesia / East Java / Surabaya	AY.23	GK
hCoV-19/Indonesia/JI-GS-ITD-51802NT/2021	EPI_ISL_5884816	12/07/2021	Asia / Indonesia / East Java / Madiun	AY.24	GK
hCoV-19/Indonesia/JI-GS-ITD-51803NT/2021	EPI_ISL_5884817	12/07/2021	Asia / Indonesia / East Java / Madiun	AY.24	GK
hCoV-19/Indonesia/JI-NIHRD-WGS09842/2021	EPI_ISL_4104532	13/07/2021	Asia / Indonesia / East Java / Nganjuk	B.1.466.2	GH
hCoV-19/Indonesia/JI-NIHRD-WGS09843/2021	EPI_ISL_4104533	13/07/2021	Asia / Indonesia / East Java / Nganjuk	AY.23	GK
hCoV-19/Indonesia/JI-NIHRD-WGS09844/2021	EPI_ISL_4104534	13/07/2021	Asia / Indonesia / East Java / Nganjuk	AY.24	GK
hCoV-19/Indonesia/JI-NIHRD-WGS09845/2021	EPI_ISL_4104535	13/07/2021	Asia / Indonesia / East Java / Nganjuk	AY.23	GK

hCoV-19/Indonesia/JI-NIHRD-WGS09846/2021	EPI_ISL_4104536	13/07/2021	Asia / Indonesia / East Java / Nganjuk	B.1.466.2	GH
hCoV-19/Indonesia/JI-GS-ITD-46798NT/2021	EPI_ISL_5884809	13/07/2021	Asia / Indonesia / East Java / Surabaya	AY.23	GK
hCoV-19/Indonesia/JI-GS-ITD-46885NT/2021	EPI_ISL_5884810	13/07/2021	Asia / Indonesia / East Java / Surabaya	AY.24	GK
hCoV-19/Indonesia/JI-GS-ITD-52472N/2021	EPI_ISL_7189555	13/07/2021	Asia / Indonesia / East Java / Ngawi	AY.23	GK
hCoV-19/Indonesia/JI-GS-ITD-52473NT/2021	EPI_ISL_7189564	13/07/2021	Asia / Indonesia / East Java / Ngawi	AY.23	GK
hCoV-19/Indonesia/JI-GS-ITD-52474NT/2021	EPI_ISL_7189574	13/07/2021	Asia / Indonesia / East Java / Ngawi	AY.23	GK
hCoV-19/Indonesia/JI-GS-ITD-52475NT/2021	EPI_ISL_7189579	13/07/2021	Asia / Indonesia / East Java / Ngawi	AY.23	GK
hCoV-19/Indonesia/JI-GS-ITD-52478NT/2021	EPI_ISL_7189585	13/07/2021	Asia / Indonesia / East Java / Ngawi	AY.23	GK
hCoV-19/Indonesia/JI-GS-ITD-52480NT/2021	EPI_ISL_7189593	13/07/2021	Asia / Indonesia / East Java / Ngawi	AY.23	GK
hCoV-19/Indonesia/JI-GS-ITD-51796NT/2021	EPI_ISL_5884811	16/07/2021	Asia / Indonesia / East Java / Malang	AY.23	GK
hCoV-19/Indonesia/JI-GS-ITD-51798NT/2021	EPI_ISL_5884815	16/07/2021	Asia / Indonesia / East Java / Malang	AY.23	GK
hCoV-19/Indonesia/JI-NIHRD-WGS09052/2021	EPI_ISL_4090583	18/07/2021	Asia / Indonesia / East Java / Magetan	AY.23	GK
hCoV-19/Indonesia/JI-PNF-214354/2021	EPI_ISL_6425638	21/07/2021	Asia / Indonesia / East Java / Surabaya	AY.24	GK
hCoV-19/Indonesia/JI-ITD-48475NT/2021	EPI_ISL_4056016	24/07/2021	Asia / Indonesia / East Java / Surabaya	AY.24	GK
hCoV-19/Indonesia/JI-NIHRD-WGS09847/2021	EPI_ISL_4104537	26/07/2021	Asia / Indonesia / East Java / Nganjuk	AY.23	G
hCoV-19/Indonesia/JI-ITD-48946NT/2021	EPI_ISL_4056017	28/07/2021	Asia / Indonesia / East Java / Surabaya	AY.24	GK
hCoV-19/Indonesia/JI-GS-ITD-51924N/2021	EPI_ISL_5884822	28/07/2021	Asia / Indonesia / East Java / Surabaya	AY.59	GK
hCoV-19/Indonesia/JI-GS-ITD-51925N/2021	EPI_ISL_5884823	28/07/2021	Asia / Indonesia / East Java / Surabaya	AY.79	GK
hCoV-19/Indonesia/JI-GS-ITD-51926N/2021	EPI_ISL_5884824	28/07/2021	Asia / Indonesia / East Java / Surabaya	AY.59	GK
hCoV-19/Indonesia/JI-GS-ITD-51927N/2021	EPI_ISL_5884825	28/07/2021	Asia / Indonesia / East Java / Surabaya	AY.59	GK
hCoV-19/Indonesia/JI-GS-ITD-51928N/2021	EPI_ISL_5884826	28/07/2021	Asia / Indonesia / East Java / Surabaya	AY.59	GK
hCoV-19/Indonesia/JI-GS-ITD-51929N/2021	EPI_ISL_5884827	28/07/2021	Asia / Indonesia / East Java / Surabaya	AY.59	GK
hCoV-19/Indonesia/JI-GS-ITD-51930N/2021	EPI_ISL_5884828	28/07/2021	Asia / Indonesia / East Java / Surabaya	B.1.617.2	GK
hCoV-19/Indonesia/JI-GS-ITD-51931N/2021	EPI_ISL_5884829	28/07/2021	Asia / Indonesia / East Java / Surabaya	AY.79	GK
hCoV-19/Indonesia/JI-NIHRD-WGS10126/2021	EPI_ISL_4254879	29/07/2021	Asia / Indonesia / East Java / Malang	B.1.466.2	GH
hCoV-19/Indonesia/JI-ITD-49337NT/2021	EPI_ISL_4742355	31/07/2021	Asia / Indonesia / East Java / Sidoarjo	AY.23	GK
hCoV-19/Indonesia/JI-NIHRD-WGS10094/2021	EPI_ISL_4254869	02/08/2021	Asia / Indonesia / East Java / Bondowoso	AY.23	GK

hCoV-19/Indonesia/JI-NIHRD-WGS10092/2021	EPI_ISL_4254867	03/08/2021	Asia / Indonesia / East Java / Bondowoso	AY.23	GK
hCoV-19/Indonesia/JI-NIHRD-WGS10093/2021	EPI_ISL_4254868	03/08/2021	Asia / Indonesia / East Java / Bondowoso	AY.23	GK
hCoV-19/Indonesia/JI-NIHRD-WGS10095/2021	EPI_ISL_4254870	03/08/2021	Asia / Indonesia / East Java / Bondowoso	AY.24	GK
hCoV-19/Indonesia/JI-GS-ITD-51932N/2021	EPI_ISL_5884830	05/08/2021	Asia / Indonesia / East Java / Surabaya	AY.59	GK
hCoV-19/Indonesia/JI-ITD-50088NT/2021	EPI_ISL_4056018	06/08/2021	Asia / Indonesia / East Java / Sidoarjo	AY.23	GK
hCoV-19/Indonesia/JI-NIHRD-WGS10124/2021	EPI_ISL_4254878	07/08/2021	Asia / Indonesia / East Java / Malang	AY.23	GK
hCoV-19/Indonesia/JI-GS-ITD-51922N/2021	EPI_ISL_5884821	07/08/2021	Asia / Indonesia / East Java / Surabaya	AY.59	GK
hCoV-19/Indonesia/JI-NIHRD-WGS10096/2021	EPI_ISL_4254871	09/08/2021	Asia / Indonesia / East Java / Bondowoso	AY.23	GK
hCoV-19/Indonesia/JI-GS-ITD-50826NT/2021	EPI_ISL_7189436	13/08/2021	Asia / Indonesia / East Java / Gresik	AY.23	GK
hCoV-19/Indonesia/JI-GS-ITD-51316N/2021	EPI_ISL_7189439	25/08/2021	Asia / Indonesia / East Java / Pasuruan	AY.24	GK
hCoV-19/Indonesia/JI-GS-ITD-51353NT/2021	EPI_ISL_7189448	26/08/2021	Asia / Indonesia / East Java / Surabaya	AY.23	GK
hCoV-19/Indonesia/JI-GS-ITD-51357NT/2021	EPI_ISL_7189452	26/08/2021	Asia / Indonesia / East Java / Sidoarjo	AY.24	GK
hCoV-19/Indonesia/JI-GS-ITD-51361NT/2021	EPI_ISL_7189460	26/08/2021	Asia / Indonesia / East Java / Sidoarjo	AY.23	GK
hCoV-19/Indonesia/JI-GS-ITD-51362NT/2021	EPI_ISL_7189465	26/08/2021	Asia / Indonesia / East Java / Sidoarjo	AY.23	GK
hCoV-19/Indonesia/JI-ITD-51418N/2021	EPI_ISL_4056019	28/08/2021	Asia / Indonesia / East Java / Surabaya	AY.24	GK
hCoV-19/Indonesia/JI-GS-ITD-51490NT/2021	EPI_ISL_7189468	29/08/2021	Asia / Indonesia / East Java / Surabaya	AY.24	GK
hCoV-19/Indonesia/JI-GS-ITD-51493NT/2021	EPI_ISL_7189477	30/08/2021	Asia / Indonesia / East Java / Surabaya	AY.24	GK
hCoV-19/Indonesia/JI-GS-ITD-51511NT/2021	EPI_ISL_7189482	31/08/2021	Asia / Indonesia / East Java / Surabaya	AY.23	GK
hCoV-19/Indonesia/JI-GS-ITD-51936N/2021	EPI_ISL_5884832	01/09/2021	Asia / Indonesia / East Java / Surabaya	B.1.617.2	GK
hCoV-19/Indonesia/JI-GS-ITD-51681NT/2021	EPI_ISL_7189492	03/09/2021	Asia / Indonesia / East Java / Surabaya	AY.23	GK
hCoV-19/Indonesia/JI-GS-ITD-51935N/2021	EPI_ISL_5884831	06/09/2021	Asia / Indonesia / East Java / Surabaya	B.1.617.2	GK
hCoV-19/Indonesia/JI-GS-ITD-51881N/2021	EPI_ISL_5884818	09/09/2021	Asia / Indonesia / East Java / Surabaya	AY.59	GK
hCoV-19/Indonesia/JI-GS-ITD-51882N/2021	EPI_ISL_5884819	09/09/2021	Asia / Indonesia / East Java / Surabaya	AY.23	GK
hCoV-19/Indonesia/JI-GS-ITD-51883NT/2021	EPI_ISL_5884820	10/09/2021	Asia / Indonesia / East Java / Surabaya	AY.24	GK
hCoV-19/Indonesia/JI-GS-ITD-51890NT/2021	EPI_ISL_7189503	10/09/2021	Asia / Indonesia / East Java / Surabaya	AY.23	GK
hCoV-19/Indonesia/JI-GS-ITD-51970N/2021	EPI_ISL_5884835	13/09/2021	Asia / Indonesia / East Java / Surabaya	AY.23	GK
hCoV-19/Indonesia/JI-GS-ITD-52029N/2021	EPI_ISL_5884836	13/09/2021	Asia / Indonesia / East Java / Surabaya	AY.59	GK

hCoV-19/Indonesia/JI-GS-ITD-51953NT/2021	EPI_ISL_7189510	13/09/2021	Asia / Indonesia / East Java / Surabaya	AY.23	GK
hCoV-19/Indonesia/JI-GS-ITD-52122NT/2021	EPI_ISL_7189520	21/09/2021	Asia / Indonesia / East Java / Surabaya	AY.23	GK
hCoV-19/Indonesia/JI-GS-ITD-52236NT/2021	EPI_ISL_7189541	27/09/2021	Asia / Indonesia / East Java / Surabaya	AY.23	GK
hCoV-19/Indonesia/JI-GS-ITD-53184NT/2021	EPI_ISL_8806068	06/10/2021	Asia / Indonesia / East Java / Surabaya	AY.24	GK
hCoV-19/Indonesia/JI-GS-ITD-52458N/2021	EPI_ISL_7189549	11/10/2021	Asia / Indonesia / East Java / Surabaya	AY.23	GK
hCoV-19/Indonesia/JI-GS-ITD-53186NT/2021	EPI_ISL_8806069	11/10/2021	Asia / Indonesia / East Java / Surabaya	AY.23	GK
hCoV-19/Indonesia/JI-GS-ITD-52728NT/2021	EPI_ISL_7189724	21/10/2021	Asia / Indonesia / East Java / Blitar	AY.23	GK
hCoV-19/Indonesia/JI-GS-ITD-53189NT/2021	EPI_ISL_8806070	21/10/2021	Asia / Indonesia / East Java / Surabaya	AY.23	GK
hCoV-19/Indonesia/JI-GS-ITD-52650NT/2021	EPI_ISL_7189680	23/10/2021	Asia / Indonesia / East Java / Surabaya	AY.79	GK
hCoV-19/Indonesia/JI-GS-ITD-52671NT/2021	EPI_ISL_7189687	23/10/2021	Asia / Indonesia / East Java / Surabaya	AY.79	GK
hCoV-19/Indonesia/JI-GS-ITD-52673NT/2021	EPI_ISL_7189698	23/10/2021	Asia / Indonesia / East Java / Surabaya	AY.79	GK
hCoV-19/Indonesia/JI-GS-ITD-52729NT/2021	EPI_ISL_7189730	23/10/2021	Asia / Indonesia / East Java / Blitar	AY.23	GK
hCoV-19/Indonesia/JI-GS-ITD-52736NT/2021	EPI_ISL_7189737	23/10/2021	Asia / Indonesia / East Java / Blitar	AY.23	GK
hCoV-19/Indonesia/JI-GS-ITD-53191NT/2021	EPI_ISL_8806079	26/10/2021	Asia / Indonesia / East Java / Surabaya	AY.100	GK
hCoV-19/Indonesia/JI-GS-ITD-52718N/2021	EPI_ISL_7189705	30/10/2021	Asia / Indonesia / East Java / Surabaya	AY.24	GK
hCoV-19/Indonesia/JI-GS-ITD-52719N/2021	EPI_ISL_7189714	30/10/2021	Asia / Indonesia / East Java / Surabaya	AY.24	GK
hCoV-19/Indonesia/JI-GS-ITD-53193NT/2021	EPI_ISL_8806074	30/10/2021	Asia / Indonesia / East Java / Surabaya	AY.24	GK
hCoV-19/Indonesia/JI-GS-ITD-52956NT/2021	EPI_ISL_8215780	12/11/2021	Asia / Indonesia / East Java / Surabaya	AY.23	GK
hCoV-19/Indonesia/JI-GS-ITD-52776N/2021	EPI_ISL_8215777	16/11/2021	Asia / Indonesia / East Java / Surabaya	AY.23	GK
hCoV-19/Indonesia/JI-GS-ITD-52958N/2021	EPI_ISL_8215781	18/11/2021	Asia / Indonesia / East Java / Surabaya	AY.23	GK
hCoV-19/Indonesia/JI-GS-ITD-52959N/2021	EPI_ISL_8215782	18/11/2021	Asia / Indonesia / East Java / Surabaya	AY.23	GK
hCoV-19/Indonesia/JI-GS-ITD-52960N/2021	EPI_ISL_8215783	19/11/2021	Asia / Indonesia / East Java / Surabaya	AY.23	GK
hCoV-19/Indonesia/JI-NIHRD-WGS13534/2021	EPI_ISL_7550151	20/11/2021	Asia / Indonesia / East Java	AY.24	GK
hCoV-19/Indonesia/JI-GS-ITD-52962N/2021	EPI_ISL_8215784	25/11/2021	Asia / Indonesia / East Java / Surabaya	AY.23	GK
hCoV-19/Indonesia/JI-GS-ITD-52847N/2021	EPI_ISL_8215778	26/11/2021	Asia / Indonesia / East Java / Surabaya	AY.23	GK
hCoV-19/Indonesia/JI-GS-ITD-52963N/2021	EPI_ISL_8215785	26/11/2021	Asia / Indonesia / East Java / Surabaya	AY.23	GK
hCoV-19/Indonesia/JI-GS-ITD-53195NT/2021	EPI_ISL_8806084	26/11/2021	Asia / Indonesia / East Java / Surabaya	AY.24	GV
hCoV-19/Indonesia/JI-GS-ITD-52953NT/2021	EPI_ISL_8216465	28/11/2021	Asia / Indonesia / East Java / Ponorogo	AY.100	GK
hCoV-19/Indonesia/JI-GS-ITD-53011NT/2021	EPI_ISL_8215791	29/11/2021	Asia / Indonesia / East Java / Surabaya	AY.23	GK
hCoV-19/Indonesia/JI-GS-ITD-53012NT/2021	EPI_ISL_8215792	29/11/2021	Asia / Indonesia / East Java / Surabaya	AY.23	GK
hCoV-19/Indonesia/JI-GS-ITD-52954NT/2021	EPI_ISL_8215779	30/11/2021	Asia / Indonesia / East Java / Ponorogo	AY.23	GK

hCoV-19/Indonesia/JI-GS-ITD-53014NT/2021	EPI_ISL_8215793	30/11/2021	Asia / Indonesia / East Java / Surabaya	AY.23	GK
hCoV-19/Indonesia/JI-GS-ITD-53199NT/2021	EPI_ISL_8806081	02/12/2021	Asia / Indonesia / East Java / Surabaya	AY.23	GK
hCoV-19/Indonesia/JI-GS-ITD-53202NT/2021	EPI_ISL_8806083	03/12/2021	Asia / Indonesia / East Java / Surabaya	AY.23	GK
hCoV-19/Indonesia/JI-GS-ITD-52983NT/2021	EPI_ISL_8215786	05/12/2021	Asia / Indonesia / East Java / Surabaya	AY.24	GK
hCoV-19/Indonesia/JI-GS-ITD-52988N/2021	EPI_ISL_8215787	06/12/2021	Asia / Indonesia / East Java / Surabaya	AY.23	GK
hCoV-19/Indonesia/JI-GS-ITD-52992NT/2021	EPI_ISL_8215788	07/12/2021	Asia / Indonesia / East Java / Surabaya	AY.23	GK
hCoV-19/Indonesia/JI-GS-ITD-52993NT/2021	EPI_ISL_8215789	07/12/2021	Asia / Indonesia / East Java / Jember	AY.23	GK
hCoV-19/Indonesia/JI-GS-ITD-52995NT/2021	EPI_ISL_8215790	07/12/2021	Asia / Indonesia / East Java / Surabaya	AY.24	GK
hCoV-19/Indonesia/JI-GS-ITD-53205NT/2021	EPI_ISL_8833483	10/12/2021	Asia / Indonesia / East Java / Surabaya	AY.24	GK
hCoV-19/Indonesia/JI-GS-ITD-53032NT/2021	EPI_ISL_8215794	13/12/2021	Asia / Indonesia / East Java / Surabaya	AY.23	GK
hCoV-19/Indonesia/JI-GS-ITD-Cs53133N/2021	EPI_ISL_8479636	14/12/2021	Asia / Indonesia / East Java / Pamekasan	AY.23	GK
hCoV-19/Indonesia/JI-GS-ITD-Cs53168NT/2021	EPI_ISL_8479641	14/12/2021	Asia / Indonesia / East Java / Malang	AY.23	GK
hCoV-19/Indonesia/JI-GS-ITD-Cs53079N/2021	EPI_ISL_8479635	16/12/2021	Asia / Indonesia / East Java / Surabaya	AY.102	GK
hCoV-19/Indonesia/JI-GS-ITD-Cs53135NT/2021	EPI_ISL_8479637	17/12/2021	Asia / Indonesia / East Java / Sidoarjo	AY.23	GK
hCoV-19/Indonesia/JI-GS-ITD-Cs53136NT/2021	EPI_ISL_8479638	17/12/2021	Asia / Indonesia / East Java / Sidoarjo	AY.23	GK
hCoV-19/Indonesia/JI-GS-ITD-Cs53137NT/2021	EPI_ISL_8479639	18/12/2021	Asia / Indonesia / East Java / Surabaya	AY.23	GK
hCoV-19/Indonesia/JI-GS-ITD-53187NT/2021	EPI_ISL_8806076	20/12/2021	Asia / Indonesia / East Java / Surabaya	AY.24	GK
hCoV-19/Indonesia/JI-GS-ITD-Cs53149N/2021	EPI_ISL_8479640	21/12/2021	Asia / Indonesia / East Java / Gresik	B.1	GH
hCoV-19/Indonesia/JI-GS-ITD-53188NT/2021	EPI_ISL_8806078	24/12/2021	Asia / Indonesia / East Java / Surabaya	AY.24	GK
hCoV-19/Indonesia/JI-GS-ITD-Cs53175NT/2021	EPI_ISL_8479642	27/12/2021	Asia / Indonesia / East Java / Surabaya	AY.23	GK
hCoV-19/Indonesia/JI-GS-ITD-53214N/2021	EPI_ISL_8806071	27/12/2021	Asia / Indonesia / East Java / Sidoarjo	AY.23	GK
hCoV-19/Indonesia/JI-GS-ITD-53174NT/2021	EPI_ISL_8284092	28/12/2021	Asia / Indonesia / East Java / Surabaya	BA.1	GRA
hCoV-19/Indonesia/JI-GS-ITD-53237NT/2021	EPI_ISL_8708945	31/12/2021	Asia / Indonesia / East Java / Malang	BA.1.1	GRA
hCoV-19/Indonesia/JI-GS-ITD-53239NT/2022	EPI_ISL_8806073	01/01/2022	Asia / Indonesia / East Java / Tulungagung	AY.59	GK

hCoV-19/Indonesia/JI-GS-ITD-53215NT/2022	EPI_ISL_8806082	01/01/2022	Asia / Indonesia / East Java / Surabaya	B.1.470	GH
hCoV-19/Indonesia/JI-GS-ITD-53213NT/2022	EPI_ISL_8708947	03/01/2022	Asia / Indonesia / East Java / Surabaya	BA.1	GRA
hCoV-19/Indonesia/JI-GS-ITD-53223NT/2022	EPI_ISL_8806072	03/01/2022	Asia / Indonesia / East Java / Pasuruan	AY.23	GK
hCoV-19/Indonesia/JI-GS-ITD-53224NT/2022	EPI_ISL_8806077	03/01/2022	Asia / Indonesia / East Java / Pasuruan	AY.23	GK
hCoV-19/Indonesia/JI-GS-ITD-53235NT/2022	EPI_ISL_8806080	05/01/2022	Asia / Indonesia / East Java / Ponorogo	AY.126	GK
hCoV-19/Indonesia/JI-GS-ITD-53392NT/2022	EPI_ISL_9733085	05/01/2022	Asia / Indonesia / East Java / Surabaya	AY.59	GK
hCoV-19/Indonesia/JI-GS-ITD-53232NT/2022	EPI_ISL_8708948	06/01/2022	Asia / Indonesia / East Java / Surabaya	BA.1.1	GRA
hCoV-19/Indonesia/JI-GS-ITD-53233NT/2022	EPI_ISL_8708949	06/01/2022	Asia / Indonesia / East Java / Surabaya	BA.1.1	GRA
hCoV-19/Indonesia/JI-GS-ITD-53234NT/2022	EPI_ISL_8708950	06/01/2022	Asia / Indonesia / East Java / Surabaya	BA.1.1	GRA
hCoV-19/Indonesia/JI-GS-ITD-53240NT/2022	EPI_ISL_8708951	07/01/2022	Asia / Indonesia / East Java / Surabaya	BA.1.1	GRA
hCoV-19/Indonesia/JI-GS-ITD-53247NT/2022	EPI_ISL_8806075	07/01/2022	Asia / Indonesia / East Java / Gresik	AY.127	GK
hCoV-19/Indonesia/JI-GS-ITD-53246NT/2022	EPI_ISL_8708946	08/01/2022	Asia / Indonesia / East Java / Malang	BA.1.1	GRA
hCoV-19/Indonesia/JI-GS-ITD-53302N/2022	EPI_ISL_9458581	08/01/2022	Asia / Indonesia / East Java / Surabaya	BA.1.1	GRA
hCoV-19/Indonesia/JI-GS-ITD-53284NT/2022	EPI_ISL_9458543	09/01/2022	Asia / Indonesia / East Java / Malang	AY.23	GK
hCoV-19/Indonesia/JI-GS-ITD-53402NT/2022	EPI_ISL_9733119	10/01/2022	Asia / Indonesia / East Java / Malang	BA.1.1	GRA
hCoV-19/Indonesia/JI-GS-ITD-53404NT/2022	EPI_ISL_9733131	10/01/2022	Asia / Indonesia / East Java / Malang	BA.1.1	GRA
hCoV-19/Indonesia/JI-GS-ITD-53285NT/2022	EPI_ISL_9458544	11/01/2022	Asia / Indonesia / East Java / Malang	B.1.617.2	GK
hCoV-19/Indonesia/JI-GS-ITD-53298NT/2022	EPI_ISL_9458552	12/01/2022	Asia / Indonesia / East Java / Malang	BA.1.1	GRA
hCoV-19/Indonesia/JI-GS-ITD-53299NT/2022	EPI_ISL_9458553	12/01/2022	Asia / Indonesia / East Java / Malang	BA.1.1	GRA
hCoV-19/Indonesia/JI-GS-ITD-53286NT/2022	EPI_ISL_9458545	14/01/2022	Asia / Indonesia / East Java / Malang	BA.1	GRA
hCoV-19/Indonesia/JI-GS-ITD-53287NT/2022	EPI_ISL_9458546	14/01/2022	Asia / Indonesia / East Java / Pasuruan	BA.1.1	GRA
hCoV-19/Indonesia/JI-GS-ITD-53288NT/2022	EPI_ISL_9458547	14/01/2022	Asia / Indonesia / East Java / Pasuruan	BA.1	GRA
hCoV-19/Indonesia/JI-GS-ITD-53289NT/2022	EPI_ISL_9458548	14/01/2022	Asia / Indonesia / East Java / Pasuruan	BA.1.1	GRA
hCoV-19/Indonesia/JI-GS-ITD-53293NT/2022	EPI_ISL_9458549	14/01/2022	Asia / Indonesia / East Java / Madiun	BA.1.1	GRA
hCoV-19/Indonesia/JI-GS-ITD-53294NT/2022	EPI_ISL_9458550	14/01/2022	Asia / Indonesia / East Java / Madiun	BA.1.1	GRA
hCoV-19/Indonesia/JI-GS-ITD-53300NT/2022	EPI_ISL_9458554	14/01/2022	Asia / Indonesia / East Java / Malang	BA.1	GRA
hCoV-19/Indonesia/JI-GS-ITD-53301NT/2022	EPI_ISL_9458555	14/01/2022	Asia / Indonesia / East Java / Malang	BA.1	GRA
hCoV-19/Indonesia/JI-GS-ITD-53335NT/2022	EPI_ISL_9458558	14/01/2022	Asia / Indonesia / East Java / Madiun	BA.1	GRA
hCoV-19/Indonesia/JI-GS-ITD-53341NT/2022	EPI_ISL_9458564	14/01/2022	Asia / Indonesia / East Java / Surabaya	BA.1	GRA
hCoV-19/Indonesia/JI-GS-ITD-53342NT/2022	EPI_ISL_9458565	14/01/2022	Asia / Indonesia / East Java / Surabaya	BA.1	GRA
hCoV-19/Indonesia/JI-GS-ITD-53344NT/2022	EPI_ISL_9458566	14/01/2022	Asia / Indonesia / East Java / Malang	BA.1.1	GRA
hCoV-19/Indonesia/JI-GS-ITD-53303N/2022	EPI_ISL_9458582	14/01/2022	Asia / Indonesia / East Java / Surabaya	AY.23	GK

hCoV-19/Indonesia/JI-Labkes-45B/2022	EPI_ISL_9319154	15/01/2022	Asia / Indonesia / East Java	AY.23	GK
hCoV-19/Indonesia/JI-GS-ITD-53304N/2022	EPI_ISL_9458583	15/01/2022	Asia / Indonesia / East Java / Surabaya	AY.24	GK
hCoV-19/Indonesia/JI-GS-ITD-53383N/2022	EPI_ISL_9733084	15/01/2022	Asia / Indonesia / East Java / Surabaya	AY.23	GK
hCoV-19/Indonesia/JI-GS-ITD-53384N/2022	EPI_ISL_9733113	15/01/2022	Asia / Indonesia / East Java / Surabaya	BA.1.1	GRA
hCoV-19/Indonesia/JI-GS-ITD-53385N/2022	EPI_ISL_9733114	15/01/2022	Asia / Indonesia / East Java / Surabaya	BA.1.1	GRA
hCoV-19/Indonesia/JI-GS-ITD-53386N/2022	EPI_ISL_9733116	15/01/2022	Asia / Indonesia / East Java / Surabaya	BA.1.1	GRA
hCoV-19/Indonesia/JI-GS-ITD-53387N/2022	EPI_ISL_9733117	15/01/2022	Asia / Indonesia / East Java / Surabaya	BA.1.1	GRA
hCoV-19/Indonesia/JI-GS-ITD-53405NT/2022	EPI_ISL_9733120	15/01/2022	Asia / Indonesia / East Java / Malang	BA.1.1	GRA
hCoV-19/Indonesia/JI-Labkes-46B/2022	EPI_ISL_9319155	16/01/2022	Asia / Indonesia / East Java	BA.1.1	GRA
hCoV-19/Indonesia/JI-GS-ITD-53283NT/2022	EPI_ISL_9458542	16/01/2022	Asia / Indonesia / East Java / Surabaya	BA.1	GRA
hCoV-19/Indonesia/JI-GS-ITD-53408NT/2022	EPI_ISL_9733103	16/01/2022	Asia / Indonesia / East Java / Malang	BA.1.1	GRA
hCoV-19/Indonesia/JI-GS-ITD-53412NT/2022	EPI_ISL_9733104	16/01/2022	Asia / Indonesia / East Java / Malang	BA.1.1	GRA
hCoV-19/Indonesia/JI-GS-ITD-53409NT/2022	EPI_ISL_9733118	16/01/2022	Asia / Indonesia / East Java / Malang	BA.1	GRA
hCoV-19/Indonesia/JI-GS-ITD-53411NT/2022	EPI_ISL_9733122	16/01/2022	Asia / Indonesia / East Java / Malang	BA.1.1	GRA
hCoV-19/Indonesia/JI-GS-ITD-53407NT/2022	EPI_ISL_9733130	16/01/2022	Asia / Indonesia / East Java / Malang	BA.1	GRA
hCoV-19/Indonesia/JI-Labkes-47B/2022	EPI_ISL_9319156	17/01/2022	Asia / Indonesia / East Java	BA.1	GRA
hCoV-19/Indonesia/JI-GS-ITD-53296NT/2022	EPI_ISL_9458551	17/01/2022	Asia / Indonesia / East Java / Surabaya	BA.1	GRA
hCoV-19/Indonesia/JI-GS-ITD-53305NT/2022	EPI_ISL_9458556	17/01/2022	Asia / Indonesia / East Java / Surabaya	BA.1.1	GRA
hCoV-19/Indonesia/JI-GS-ITD-53308NT/2022	EPI_ISL_9458557	17/01/2022	Asia / Indonesia / East Java / Malang	BA.1.1	GRA
hCoV-19/Indonesia/JI-GS-ITD-53336NT/2022	EPI_ISL_9458559	17/01/2022	Asia / Indonesia / East Java / Malang	BA.1	GRA
hCoV-19/Indonesia/JI-GS-ITD-53337NT/2022	EPI_ISL_9458560	17/01/2022	Asia / Indonesia / East Java / Surabaya	BA.1	GRA
hCoV-19/Indonesia/JI-GS-ITD-53338NT/2022	EPI_ISL_9458561	17/01/2022	Asia / Indonesia / East Java / Surabaya	BA.1	GRA
hCoV-19/Indonesia/JI-GS-ITD-53339NT/2022	EPI_ISL_9458562	17/01/2022	Asia / Indonesia / East Java / Surabaya	BA.1	GRA
hCoV-19/Indonesia/JI-GS-ITD-53340NT/2022	EPI_ISL_9458563	17/01/2022	Asia / Indonesia / East Java / Surabaya	BA.1.1	GRA
hCoV-19/Indonesia/JI-GS-ITD-53355NT/2022	EPI_ISL_9458575	17/01/2022	Asia / Indonesia / East Java / Surabaya	BA.1.1	GRA
hCoV-19/Indonesia/JI-GS-ITD-53441NT/2022	EPI_ISL_9733105	17/01/2022	Asia / Indonesia / East Java / Madiun	BA.1.1	GRA
hCoV-19/Indonesia/JI-GS-ITD-53436NT/2022	EPI_ISL_9733110	17/01/2022	Asia / Indonesia / East Java / Malang	BA.1.1	GRA
hCoV-19/Indonesia/JI-GS-ITD-53345NT/2022	EPI_ISL_9458567	18/01/2022	Asia / Indonesia / East Java / Malang	BA.1.1	GRA
hCoV-19/Indonesia/JI-GS-ITD-53346NT/2022	EPI_ISL_9458568	18/01/2022	Asia / Indonesia / East Java / Malang	BA.1	GRA
hCoV-19/Indonesia/JI-GS-ITD-53347NT/2022	EPI_ISL_9458569	18/01/2022	Asia / Indonesia / East Java / Malang	BA.1.1	GRA
hCoV-19/Indonesia/JI-GS-ITD-53348NT/2022	EPI_ISL_9458570	18/01/2022	Asia / Indonesia / East Java / Malang	BA.1	GRA
hCoV-19/Indonesia/JI-GS-ITD-53349NT/2022	EPI_ISL_9458571	18/01/2022	Asia / Indonesia / East Java / Surabaya	BA.1	GRA

hCoV-19/Indonesia/JI-GS-ITD-53352NT/2022	EPI_ISL_9458572	18/01/2022	Asia / Indonesia / East Java / Surabaya	BA.1.1	GRA
hCoV-19/Indonesia/JI-GS-ITD-53353NT/2022	EPI_ISL_9458573	18/01/2022	Asia / Indonesia / East Java / Surabaya	BA.1.1	GRA
hCoV-19/Indonesia/JI-GS-ITD-53354NT/2022	EPI_ISL_9458574	18/01/2022	Asia / Indonesia / East Java / Surabaya	BA.1.1	GRA
hCoV-19/Indonesia/JI-GS-ITD-53357NT/2022	EPI_ISL_9458576	18/01/2022	Asia / Indonesia / East Java / Madiun	BA.1	GRA
hCoV-19/Indonesia/JI-GS-ITD-53358NT/2022	EPI_ISL_9458577	18/01/2022	Asia / Indonesia / East Java / Surabaya	BA.1	GRA
hCoV-19/Indonesia/JI-GS-ITD-53368NT/2022	EPI_ISL_9458580	18/01/2022	Asia / Indonesia / East Java / Lamongan	BA.1.1	GRA
hCoV-19/Indonesia/JI-GS-ITD-53307N/2022	EPI_ISL_9458584	18/01/2022	Asia / Indonesia / East Java / Mojokerto	BA.1.1	GRA
hCoV-19/Indonesia/JI-GS-ITD-53364N/2022	EPI_ISL_9458585	18/01/2022	Asia / Indonesia / East Java / Sampang	BA.1	GRA
hCoV-19/Indonesia/JI-GS-ITD-53380N/2022	EPI_ISL_9733086	18/01/2022	Asia / Indonesia / East Java / Sidoarjo	BA.2	GRA
hCoV-19/Indonesia/JI-GS-ITD-53369NT/2022	EPI_ISL_9733088	18/01/2022	Asia / Indonesia / East Java / Surabaya	BA.1	GRA
hCoV-19/Indonesia/JI-GS-ITD-53410NT/2022	EPI_ISL_9733121	18/01/2022	Asia / Indonesia / East Java / Malang	BA.1.1	GRA
hCoV-19/Indonesia/JI-GS-ITD-53406NT/2022	EPI_ISL_9733123	18/01/2022	Asia / Indonesia / East Java / Malang	BA.1.1	GRA
hCoV-19/Indonesia/JI-GS-ITD-53401NT/2022	EPI_ISL_9733125	18/01/2022	Asia / Indonesia / East Java / Malang	BA.1.1	GRA
hCoV-19/Indonesia/JI-GS-ITD-53362NT/2022	EPI_ISL_9458578	19/01/2022	Asia / Indonesia / East Java / Surabaya	BA.1.1	GRA
hCoV-19/Indonesia/JI-GS-ITD-53367NT/2022	EPI_ISL_9458579	19/01/2022	Asia / Indonesia / East Java / Surabaya	BA.1.1	GRA
hCoV-19/Indonesia/JI-GS-ITD-53366N/2022	EPI_ISL_9458586	19/01/2022	Asia / Indonesia / East Java / Surabaya	BA.1	GRA
hCoV-19/Indonesia/JI-GS-ITD-53370NT/2022	EPI_ISL_9733089	19/01/2022	Asia / Indonesia / East Java / Surabaya	BA.1	GRA
hCoV-19/Indonesia/JI-GS-ITD-53376NT/2022	EPI_ISL_9733092	19/01/2022	Asia / Indonesia / East Java / Surabaya	BA.1.1	GRA
hCoV-19/Indonesia/JI-GS-ITD-53379NT/2022	EPI_ISL_9733093	19/01/2022	Asia / Indonesia / East Java / Surabaya	BA.1	GRA
hCoV-19/Indonesia/JI-GS-ITD-53396NT/2022	EPI_ISL_9733094	19/01/2022	Asia / Indonesia / East Java / Surabaya	BA.1	GRA
hCoV-19/Indonesia/JI-GS-ITD-53416NT/2022	EPI_ISL_9733106	19/01/2022	Asia / Indonesia / East Java / Surabaya	BA.1.1	GRA
hCoV-19/Indonesia/JI-GS-ITD-53417NT/2022	EPI_ISL_9733107	19/01/2022	Asia / Indonesia / East Java / Surabaya	BA.1	GRA
hCoV-19/Indonesia/JI-GS-ITD-53394NT/2022	EPI_ISL_9733108	19/01/2022	Asia / Indonesia / East Java / Surabaya	BA.1	GRA
hCoV-19/Indonesia/JI-GS-ITD-53395NT/2022	EPI_ISL_9733109	19/01/2022	Asia / Indonesia / East Java / Surabaya	BA.1	GRA
hCoV-19/Indonesia/JI-GS-ITD-53374NT/2022	EPI_ISL_9733111	19/01/2022	Asia / Indonesia / East Java / Malang	BA.1	GRA
hCoV-19/Indonesia/JI-GS-ITD-53377NT/2022	EPI_ISL_9733112	19/01/2022	Asia / Indonesia / East Java / Surabaya	BA.1.1	GRA
hCoV-19/Indonesia/JI-GS-ITD-53378NT/2022	EPI_ISL_9733127	19/01/2022	Asia / Indonesia / East Java / Surabaya	BA.1.1	GRA
hCoV-19/Indonesia/JI-GS-ITD-53419NT/2022	EPI_ISL_9733128	19/01/2022	Asia / Indonesia / East Java / Malang	BA.1.1	GRA
hCoV-19/Indonesia/JI-GS-ITD-53372NT/2022	EPI_ISL_9778095	19/01/2022	Asia / Indonesia / East Java / Surabaya	BA.1	GRA
hCoV-19/Indonesia/JI-GS-ITD-53391NT/2022	EPI_ISL_9733090	20/01/2022	Asia / Indonesia / East Java / Surabaya	BA.1	GRA
hCoV-19/Indonesia/JI-GS-ITD-53421NT/2022	EPI_ISL_9733091	20/01/2022	Asia / Indonesia / East Java / Surabaya	BA.1	GRA
hCoV-19/Indonesia/JI-GS-ITD-53397NT/2022	EPI_ISL_9733095	20/01/2022	Asia / Indonesia / East Java / Surabaya	BA.1	GRA

hCoV-19/Indonesia/JI-GS-ITD-53398NT/2022	EPI_ISL_9733099	20/01/2022	Asia / Indonesia / East Java / Surabaya	BA.1.1	GRA
hCoV-19/Indonesia/JI-GS-ITD-53399N/2022	EPI_ISL_9733100	20/01/2022	Asia / Indonesia / East Java / Surabaya	BA.1.1	GRA
hCoV-19/Indonesia/JI-GS-ITD-53400NT/2022	EPI_ISL_9733101	20/01/2022	Asia / Indonesia / East Java / Surabaya	BA.1.1	GRA
hCoV-19/Indonesia/JI-GS-ITD-53427NT/2022	EPI_ISL_9733096	21/01/2022	Asia / Indonesia / East Java / Surabaya	BA.1	GRA
hCoV-19/Indonesia/JI-GS-ITD-53428NT/2022	EPI_ISL_9733097	21/01/2022	Asia / Indonesia / East Java / Surabaya	BA.1	GRA
hCoV-19/Indonesia/JI-GS-ITD-53429NT/2022	EPI_ISL_9733098	21/01/2022	Asia / Indonesia / East Java / Surabaya	BA.1	GRA
hCoV-19/Indonesia/JI-GS-ITD-53433N/2022	EPI_ISL_9733087	22/01/2022	Asia / Indonesia / East Java / Jember	BA.1.1	GRA
hCoV-19/Indonesia/JI-GS-ITD-53438NT/2022	EPI_ISL_9733102	22/01/2022	Asia / Indonesia / East Java / Surabaya	BA.1	GRA
hCoV-19/Indonesia/JI-GS-ITD-53446NT/2022	EPI_ISL_9733124	22/01/2022	Asia / Indonesia / East Java / Surabaya	BA.1	GRA
hCoV-19/Indonesia/JI-GS-ITD-53443NT/2022	EPI_ISL_9733126	22/01/2022	Asia / Indonesia / East Java / Surabaya	AY.59	GK
Virus name	Accession ID	Collection date	Location	Lineage	Clade
hCoV-19/Indonesia/JI-ITD-136N/2020	EPI_ISL_529961	12/03/2020	Asia / Indonesia / East Java / Surabaya	B	L
hCoV-19/Indonesia/JI-ITD-150Sp/2020	EPI_ISL_529962	16/03/2020	Asia / Indonesia / East Java / Surabaya	B	L
hCoV-19/Indonesia/JI-ITD-853Sp/2020	EPI_ISL_437187	25/03/2020	Asia / Indonesia / East Java / Surabaya	B.50	L
hCoV-19/Indonesia/JI-ITDua-966NTv/2020	EPI_ISL_759966	26/03/2020	Asia / Indonesia / East Java / Sidoarjo	B	L
hCoV-19/Indonesia/JI-ITD-1038NTv/2020	EPI_ISL_2284873	27/03/2020	Asia / Indonesia / East Java / Surabaya	B.1	G
hCoV-19/Indonesia/JI-ITDua-1006NTv/2020	EPI_ISL_759961	27/03/2020	Asia / Indonesia / East Java / Surabaya	B.56	L
hCoV-19/Indonesia/JI-ITD-1238Sp/2020	EPI_ISL_458079	30/03/2020	Asia / Indonesia / East Java / Surabaya	B.56	L
hCoV-19/Indonesia/JI-ITD-1273NT/2020	EPI_ISL_458081	30/03/2020	Asia / Indonesia / East Java / Pasuruan	B	L
hCoV-19/Indonesia/JI-ITD-1273V/2020	EPI_ISL_529964	30/03/2020	Asia / Indonesia / East Java / Pasuruan	B	L
hCoV-19/Indonesia/JI-ITDua-998NTv/2020	EPI_ISL_759959	30/03/2020	Asia / Indonesia / East Java / Surabaya	B.56	L
hCoV-19/Indonesia/JI-ITDua-1609Nv/2020	EPI_ISL_759967	01/04/2020	Asia / Indonesia / East Java / Tulungagung	B.1	GH
hCoV-19/Indonesia/JI-ITD-2766NT/2020	EPI_ISL_458082	09/04/2020	Asia / Indonesia / East Java / Surabaya	B.1	GH
hCoV-19/Indonesia/JI-ITDua-2858NTv/2020	EPI_ISL_759965	09/04/2020	Asia / Indonesia / East Java / Surabaya	B.1.470	GH
hCoV-19/Indonesia/JI-ITD-3101NT/2020	EPI_ISL_458083	11/04/2020	Asia / Indonesia / East Java / Surabaya	B	L
hCoV-19/Indonesia/JI-ITD-3590NT/2020	EPI_ISL_437188	14/04/2020	Asia / Indonesia / East Java / Surabaya	B.1.470	GH
hCoV-19/Indonesia/JI-ITD-3601NT/2020	EPI_ISL_560991	14/04/2020	Asia / Indonesia / East Java / Surabaya	B.6	O
hCoV-19/Indonesia/JI-ITDua-4134NTv/2020	EPI_ISL_759962	17/04/2020	Asia / Indonesia / East Java / Surabaya	B.1.470	GH
hCoV-19/Indonesia/JI-ITDua-4437NTv/2020	EPI_ISL_759955	19/04/2020	Asia / Indonesia / East Java / Surabaya	B.1	GH
hCoV-19/Indonesia/JI-ITD-4859V/2020	EPI_ISL_529965	22/04/2020	Asia / Indonesia / East Java / Surabaya	B.1.470	GH
hCoV-19/Indonesia/JI-ITDua-5235NTv/2020	EPI_ISL_759956	25/04/2020	Asia / Indonesia / East Java / Surabaya	B.1	GH
hCoV-19/Indonesia/JI-ITDua-5392NTv/2020	EPI_ISL_759957	27/04/2020	Asia / Indonesia / East Java / Sidoarjo	B.1.470	GH

hCoV-19/Indonesia/JI-ITDua-5748NTv/2020	EPI_ISL_759958	29/04/2020	Asia / Indonesia / East Java / Surabaya	B.1.470	GH
hCoV-19/Indonesia/JI-ITDua-6033NTv/2020	EPI_ISL_759964	30/04/2020	Asia / Indonesia / East Java / Sidoarjo	B.1	GH
hCoV-19/Indonesia/JI-GSI-P3SCSBY/2020	EPI_ISL_872897	04/05/2020	Asia / Indonesia / East Java	None	O
hCoV-19/Indonesia/JI-ITDua-6647NTv/2020	EPI_ISL_956307	04/05/2020	Asia / Indonesia / East Java / Surabaya	B.1.1	GR
hCoV-19/Indonesia/JI-ITD-7061V/2020	EPI_ISL_529966	05/05/2020	Asia / Indonesia / East Java / Sidoarjo	B.1	GH
hCoV-19/Indonesia/JI-ITD-8402NT/2020	EPI_ISL_529138	11/05/2020	Asia / Indonesia / East Java / Surabaya	B.1	GH
hCoV-19/Indonesia/JI-ITDua-8967NTv/2020	EPI_ISL_956270	12/05/2020	Asia / Indonesia / East Java / Surabaya	B.1.470	GH
hCoV-19/Indonesia/JI-ITDua-9627NTv/2020	EPI_ISL_956308	14/05/2020	Asia / Indonesia / East Java / Surabaya	B.1	GH
hCoV-19/Indonesia/JI-ITDua-11000NTv/2020	EPI_ISL_956271	18/05/2020	Asia / Indonesia / East Java / Surabaya	B.1.470	GH
hCoV-19/Indonesia/JI-RSDS-RCVTD-UNAIR-6B/2020	EPI_ISL_1366083	19/05/2020	Asia / Indonesia / East Java	B.1.470	GH
hCoV-19/Indonesia/JI-RSDS-RCVTD-UNAIR-11-B/2020	EPI_ISL_1366238	19/05/2020	Asia / Indonesia / East Java / Surabaya	B.1.470	G
hCoV-19/Indonesia/JI-ITDua-12009NTv/2020	EPI_ISL_956273	23/05/2020	Asia / Indonesia / East Java / Surabaya	B.1.470	GH
hCoV-19/Indonesia/JI-ITD-12202V/2020	EPI_ISL_529967	09/06/2020	Asia / Indonesia / East Java / Sidoarjo	B.1.470	GH
hCoV-19/Indonesia/JI-ITDua-12323Nvv/2020	EPI_ISL_759963	09/06/2020	Asia / Indonesia / East Java / Sidoarjo	B.1.470	GH
hCoV-19/Indonesia/JI-ITDua-12418Nv/2020	EPI_ISL_956309	10/06/2020	Asia / Indonesia / East Java / Sidoarjo	B.1.470	GH
hCoV-19/Indonesia/JI-NIHRD-PME0999/2020	EPI_ISL_538498	11/06/2020	Asia / Indonesia / East Java	B.1.470	GH
hCoV-19/Indonesia/JI-ITDua-12663Nv/2020	EPI_ISL_956274	13/06/2020	Asia / Indonesia / East Java / Sidoarjo	B.1.456	GH
hCoV-19/Indonesia/JI-RSDS-RCVTD-UNAIR-35-A/2020	EPI_ISL_1364466	24/06/2020	Asia / Indonesia / East Java	B.1	GH
hCoV-19/Indonesia/JI-RSDS-RCVTD-UNAIR-35-B/2020	EPI_ISL_1364467	24/06/2020	Asia / Indonesia / East Java	B.1	GH
hCoV-19/Indonesia/JI-RSDS-RCVTD-UNAIR-35-C/2020	EPI_ISL_1364468	24/06/2020	Asia / Indonesia / East Java	B.1.470	GH
hCoV-19/Indonesia/RSDS-RCVTD-UNAIR-35-C/2020	EPI_ISL_1366269	24/06/2020	Asia / Indonesia / East Java / Surabaya	B.1.470	GH
hCoV-19/Indonesia/JI-RSDS-RCVTD-UNAIR-33-B/2020	EPI_ISL_1366271	24/06/2020	Asia / Indonesia / East Java / Surabaya	B.1	GH
hCoV-19/Indonesia/JI-RSDS-RCVTD-UNAIR-33-C/2020	EPI_ISL_1366273	24/06/2020	Asia / Indonesia / East Java / Surabaya	B.1	GH

hCoV-19/Indonesia/JI-ITDua-13686NTv/2020	EPI_ISL_956276	24/06/2020	Asia / Indonesia / East Java / Sidoarjo	B.1.470	GH
hCoV-19/Indonesia/JI-NIHRD-PME2054/2020	EPI_ISL_538499	29/06/2020	Asia / Indonesia / East Java	B.1.470	GH
hCoV-19/Indonesia/JI-ITDua-16792Nv/2020	EPI_ISL_759960	14/07/2020	Asia / Indonesia / East Java / Surabaya	B.1	GH
hCoV-19/Indonesia/JI-ITDua-16761NTv/2020	EPI_ISL_956310	14/07/2020	Asia / Indonesia / East Java / Surabaya	B.1.470	GH
hCoV-19/Indonesia/JI-ITDua-17047NTv/2020	EPI_ISL_956311	16/07/2020	Asia / Indonesia / East Java / Surabaya	B.1.36.19	GH
hCoV-19/Indonesia/JI-ITD-17398NT/2020	EPI_ISL_529963	18/07/2020	Asia / Indonesia / East Java / Surabaya	B.1.470	GH
hCoV-19/Indonesia/JI-ITDua-17563Nv/2020	EPI_ISL_956277	20/07/2020	Asia / Indonesia / East Java / Surabaya	B.1.470	GH
hCoV-19/Indonesia/JI-ITDua-18214NTv/2020	EPI_ISL_956278	24/07/2020	Asia / Indonesia / East Java / Sidoarjo	B.1.470	GH
hCoV-19/Indonesia/JI-NIHRD-PME5011/2020	EPI_ISL_538511	01/08/2020	Asia / Indonesia / East Java	B.1.456	GH
hCoV-19/Indonesia/JI-ITD-19903NTv/2020	EPI_ISL_1165067	03/08/2020	Asia / Indonesia / East Java / Sidoarjo	B.1.470	GH
hCoV-19/Indonesia/JI-ITD-19882Nv/2020	EPI_ISL_1165075	03/08/2020	Asia / Indonesia / East Java / Surabaya	B.1	GH
hCoV-19/Indonesia/JI-ITD-21907NTv/2020	EPI_ISL_1165068	13/08/2020	Asia / Indonesia / East Java / Sidoarjo	B.1	GH
hCoV-19/Indonesia/JI-ITD-24254NTv/2020	EPI_ISL_1165069	28/08/2020	Asia / Indonesia / East Java / Sidoarjo	B.1	GH
hCoV-19/Indonesia/JI-ITD-25302Nv/2020	EPI_ISL_1159381	04/09/2020	Asia / Indonesia / East Java / Surabaya	B.1	GH
hCoV-19/Indonesia/JI-ITD-25612Nv/2020	EPI_ISL_1165076	05/09/2020	Asia / Indonesia / East Java / Sidoarjo	B.1	GH
hCoV-19/Indonesia/JI-ITD-26463NTv/2020	EPI_ISL_1165070	12/09/2020	Asia / Indonesia / East Java / Sidoarjo	B.1.1.28	GR
hCoV-19/Indonesia/JI-ITD-26529NTv/2020	EPI_ISL_1165071	14/09/2020	Asia / Indonesia / East Java / Sidoarjo	B.1.470	GH
hCoV-19/Indonesia/JI-ITDua-28257NT/2020	EPI_ISL_759968	02/10/2020	Asia / Indonesia / East Java / Surabaya	B.1.470	GH
hCoV-19/Indonesia/JI-NIHRD-PME10137/2020	EPI_ISL_791989	03/10/2020	Asia / Indonesia / East Java	B.1.1	GR
hCoV-19/Indonesia/JI-ITD-28533NTv/2020	EPI_ISL_1789113	06/10/2020	Asia / Indonesia / East Java / Sidoarjo	B.1	GH
hCoV-19/Indonesia/JI-ITD-28656Nv/2020	EPI_ISL_1159382	07/10/2020	Asia / Indonesia / East Java / Surabaya	B.1.470	GH
hCoV-19/Indonesia/JI-ITD-28759NTv/2020	EPI_ISL_1789114	09/10/2020	Asia / Indonesia / East Java / Sidoarjo	B.1.470	GH
hCoV-19/Indonesia/JI-ITD-29089NTv/2020	EPI_ISL_1159383	14/10/2020	Asia / Indonesia / East Java / Sidoarjo	B.1	GH
hCoV-19/Indonesia/JI-ITD-29375NTv/2020	EPI_ISL_1159384	19/10/2020	Asia / Indonesia / East Java / Surabaya	B.1.1.398	GR
hCoV-19/Indonesia/JI-ITD-29881NTv/2020	EPI_ISL_1159385	26/10/2020	Asia / Indonesia / East Java / Surabaya	B.1	GH
hCoV-19/Indonesia/JI-ITD-30338NTv/2020	EPI_ISL_1159386	03/11/2020	Asia / Indonesia / East Java / Sidoarjo	B.1.36.19	GH
hCoV-19/Indonesia/JI-ITD-30604Nv/2020	EPI_ISL_1159387	09/11/2020	Asia / Indonesia / East Java / Surabaya	B.1.470	GH
hCoV-19/Indonesia/JI-NIHRD-PME9750/2020	EPI_ISL_791980	09/11/2020	Asia / Indonesia / East Java	B.1.456	GH

hCoV-19/Indonesia/JI-ITD-30833Nv/2020	EPI_ISL_1159388	13/11/2020	Asia / Indonesia / East Java / Surabaya	B.1.470	GH
hCoV-19/Indonesia/JI-ITD-31235NTv/2020	EPI_ISL_1159389	20/11/2020	Asia / Indonesia / East Java / Surabaya	B.1.36.19	GH
hCoV-19/Indonesia/JI-ITD-31548NTv/2020	EPI_ISL_1789115	24/11/2020	Asia / Indonesia / East Java / Sidoarjo	B.1.36.19	GH
hCoV-19/Indonesia/JI-ITD-32011NTv/2020	EPI_ISL_2274436	30/11/2020	Asia / Indonesia / East Java / Sidoarjo	B.1.36.19	GH
hCoV-19/Indonesia/JI-ITD-32072NTv/2020	EPI_ISL_2284874	30/11/2020	Asia / Indonesia / East Java / Sidoarjo	B.1.456	GH
hCoV-19/Indonesia/JI-ITD-32113NTv/2020	EPI_ISL_1789116	01/12/2020	Asia / Indonesia / East Java / Sidoarjo	B.1.1.398	GR
hCoV-19/Indonesia/JI-ITD-32118NTv/2020	EPI_ISL_1795053	01/12/2020	Asia / Indonesia / East Java / Sidoarjo	B.1.470	GH
hCoV-19/Indonesia/JI-ITD-32173NTv/2020	EPI_ISL_1795054	01/12/2020	Asia / Indonesia / East Java / Surabaya	B.1.470	GH
hCoV-19/Indonesia/JI-ITD-32681NTv/2020	EPI_ISL_1795055	07/12/2020	Asia / Indonesia / East Java / Gresik	B.1.470	GH
hCoV-19/Indonesia/JI-ITD-33091Nv/2020	EPI_ISL_1795056	08/12/2020	Asia / Indonesia / East Java / Surabaya	B.1.456	GH
hCoV-19/Indonesia/JI-ITD-33120NTv/2020	EPI_ISL_1795057	09/12/2020	Asia / Indonesia / East Java / Gresik	B.1.470	GH
hCoV-19/Indonesia/JI-ITDua-33281NT/2020	EPI_ISL_877419	11/12/2020	Asia / Indonesia / East Java / Sidoarjo	B.1.466.2	GH
hCoV-19/Indonesia/JI-ITDua-33304NT/2020	EPI_ISL_877420	11/12/2020	Asia / Indonesia / East Java / Surabaya	B.1.470	GH
hCoV-19/Indonesia/JI-ITDua-33312NT/2020	EPI_ISL_877421	11/12/2020	Asia / Indonesia / East Java / Surabaya	B.1.470	GH
hCoV-19/Indonesia/JI-ITDua-33753N/2020	EPI_ISL_877422	15/12/2020	Asia / Indonesia / East Java / Sidoarjo	B.1.470	GH
hCoV-19/Indonesia/JI-ITDua-33816NT/2020	EPI_ISL_877423	16/12/2020	Asia / Indonesia / East Java / Surabaya	B.1.470	GH
hCoV-19/Indonesia/JI-ITDua-33807NT/2020	EPI_ISL_877456	16/12/2020	Asia / Indonesia / East Java / Surabaya	B.1.466.2	GH
hCoV-19/Indonesia/JI-ITDua-33817NT/2020	EPI_ISL_877457	16/12/2020	Asia / Indonesia / East Java / Surabaya	B.1.459	GH
hCoV-19/Indonesia/JI-ITDua-33862NT/2020	EPI_ISL_877458	16/12/2020	Asia / Indonesia / East Java / Sidoarjo	B.1.468	GH
hCoV-19/Indonesia/JI-ITDua-34192NT/2020	EPI_ISL_877459	18/12/2020	Asia / Indonesia / East Java / Surabaya	B.1.470	GH
hCoV-19/Indonesia/JI-ITDua-34158NT/2020	EPI_ISL_882769	18/12/2020	Asia / Indonesia / East Java / Surabaya	B.1.456	GH
hCoV-19/Indonesia/JI-ITDua-34660NT/2020	EPI_ISL_877460	22/12/2020	Asia / Indonesia / East Java / Sidoarjo	B.1.459	GH
hCoV-19/Indonesia/JI-ITDua-34618NT/2020	EPI_ISL_882770	22/12/2020	Asia / Indonesia / East Java / Surabaya	B.1.470	GH
hCoV-19/Indonesia/JI-ITD-34777NTv/2020	EPI_ISL_1795058	23/12/2020	Asia / Indonesia / East Java / Surabaya	B.1.470	GH
hCoV-19/Indonesia/JI-ITDua-34962NT/2020	EPI_ISL_888591	24/12/2020	Asia / Indonesia / East Java / Sidoarjo	B.1.470	GH
hCoV-19/Indonesia/JI-ITDua-34965NT/2020	EPI_ISL_888593	24/12/2020	Asia / Indonesia / East Java / Sidoarjo	B.1.470	GH
hCoV-19/Indonesia/JI-ITDua-35379NT/2020	EPI_ISL_877538	28/12/2020	Asia / Indonesia / East Java / Sidoarjo	B.1.470	GH
hCoV-19/Indonesia/JI-ITDua-35340NT/2020	EPI_ISL_882771	28/12/2020	Asia / Indonesia / East Java / Sidoarjo	B.1.470	GH
hCoV-19/Indonesia/JI-ITD-35608Nv/2020	EPI_ISL_1795059	29/12/2020	Asia / Indonesia / East Java / Sidoarjo	B.1.470	GH
hCoV-19/Indonesia/JI-ITDua-35767NT/2020	EPI_ISL_956279	30/12/2020	Asia / Indonesia / East Java / Sidoarjo	B.1.470	GH
hCoV-19/Indonesia/JI-ITD-35800NTv/2020	EPI_ISL_1795060	31/12/2020	Asia / Indonesia / East Java / Surabaya	B.1.110	GH
hCoV-19/Indonesia/JI-ITDua-35937NT/2021	EPI_ISL_956312	01/01/2021	Asia / Indonesia / East Java / Surabaya	B.1.470	GH

hCoV-19/Indonesia/JI-ITD-36311NTv/2021	EPI_ISL_1789117	04/01/2021	Asia / Indonesia / East Java / Sidoarjo	B.1.470	GH
hCoV-19/Indonesia/JI-ITDua-36208NT/2021	EPI_ISL_956280	04/01/2021	Asia / Indonesia / East Java / Surabaya	B.1.470	GH
hCoV-19/Indonesia/JI-ITDua-36471NT/2021	EPI_ISL_956281	04/01/2021	Asia / Indonesia / East Java / Sidoarjo	B.1.470	GH
hCoV-19/Indonesia/JI-ITDua-36484NT/2021	EPI_ISL_956315	04/01/2021	Asia / Indonesia / East Java / Sidoarjo	B.1.470	GH
hCoV-19/Indonesia/JI-ITDua-36613NT/2021	EPI_ISL_956316	06/01/2021	Asia / Indonesia / East Java / Surabaya	B.1.470	GH
hCoV-19/Indonesia/JI-ITDua-36824NT/2021	EPI_ISL_956313	08/01/2021	Asia / Indonesia / East Java / Sidoarjo	B.1.36.19	GH
hCoV-19/Indonesia/JI-ITDua-37008N/2021	EPI_ISL_956314	11/01/2021	Asia / Indonesia / East Java / Surabaya	B.1.470	GH
hCoV-19/Indonesia/JI-RSDS-RCVTD-UNAIR-54-A/2021	EPI_ISL_1366503	12/01/2021	Asia / Indonesia / East Java / Surabaya	B.1	GH
hCoV-19/Indonesia/JI-ITD-37255NT/2021	EPI_ISL_1165072	14/01/2021	Asia / Indonesia / East Java / Surabaya	B.1.466.2	GH
hCoV-19/Indonesia/JI-ITD-37338NT/2021	EPI_ISL_1165073	14/01/2021	Asia / Indonesia / East Java / Surabaya	B.1.456	GH
hCoV-19/Indonesia/JI-ITD-37257NTv/2021	EPI_ISL_1789118	14/01/2021	Asia / Indonesia / East Java / Surabaya	B.1.470	GH
hCoV-19/Indonesia/JI-ITD-37619NTv/2021	EPI_ISL_1789119	18/01/2021	Asia / Indonesia / East Java / Surabaya	B.1.466.2	GH
hCoV-19/Indonesia/JI-RSDS-RCVTD-UNAIR-49-A/2021	EPI_ISL_1366505	21/01/2021	Asia / Indonesia / East Java / Surabaya	B.1	GH
hCoV-19/Indonesia/JI-RSDS-RCVTD-UNAIR-42-A/2021	EPI_ISL_1366509	21/01/2021	Asia / Indonesia / East Java / Surabaya	B.1	GH
hCoV-19/Indonesia/JI-ITD-38076NTv/2021	EPI_ISL_1789172	22/01/2021	Asia / Indonesia / East Java / Surabaya	B.1.470	GH
hCoV-19/Indonesia/JI-ITD-38279NT/2021	EPI_ISL_1159390	25/01/2021	Asia / Indonesia / East Java / Gresik	B.1	GH
hCoV-19/Indonesia/JI-ITD-38281N/2021	EPI_ISL_1789112	25/01/2021	Asia / Indonesia / East Java / Pasuruan	B.1.470	GH
hCoV-19/Indonesia/JI-ITD-38310Nv/2021	EPI_ISL_1789202	25/01/2021	Asia / Indonesia / East Java / Surabaya	B.1.36.19	GH
hCoV-19/Indonesia/JI-ITD-38912NT/2021	EPI_ISL_1165074	02/02/2021	Asia / Indonesia / East Java / Surabaya	B.1.470	GH
hCoV-19/Indonesia/JI-ITD-39066NTv/2021	EPI_ISL_2284875	03/02/2021	Asia / Indonesia / East Java / Surabaya	B.1.470	GH
hCoV-19/Indonesia/JI-NIHRD-WGS01173/2021	EPI_ISL_1469246	04/02/2021	Asia / Indonesia / East Java	B.1.470	GH
hCoV-19/Indonesia/JI-NIHRD-WGS01175/2021	EPI_ISL_1469271	04/02/2021	Asia / Indonesia / East Java	B.1.470	GH
hCoV-19/Indonesia/JI-NIHRD-WGS01179/2021	EPI_ISL_1469272	08/02/2021	Asia / Indonesia / East Java	B.1.466.2	GH
hCoV-19/Indonesia/JI-ITD-39254NTv/2021	EPI_ISL_2284876	08/02/2021	Asia / Indonesia / East Java / Surabaya	B.1	GH
hCoV-19/Indonesia/JI-ITD-39376NT/2021	EPI_ISL_1159391	09/02/2021	Asia / Indonesia / East Java / Surabaya	B.1.470	GH
hCoV-19/Indonesia/JI-PNF-211352/2021	EPI_ISL_6425650	10/02/2021	Asia / Indonesia / East Java / Surabaya	B.1.470	GH

hCoV-19/Indonesia/JI-PNF-211373/2021	EPI_ISL_6425649	11/02/2021	Asia / Indonesia / East Java / Sidoarjo	B.1.470	GH
hCoV-19/Indonesia/JI-ITD-39660NTv/2021	EPI_ISL_2284877	15/02/2021	Asia / Indonesia / East Java / Surabaya	B.1.466.2	GH
hCoV-19/Indonesia/JI-NIHRD-WGS01181/2021	EPI_ISL_1469273	16/02/2021	Asia / Indonesia / East Java	B.1.459	GH
hCoV-19/Indonesia/JI-NIHRD-WGS01183/2021	EPI_ISL_1469274	16/02/2021	Asia / Indonesia / East Java	B.1.1.398	GR
hCoV-19/Indonesia/JI-NIHRD-WGS01184/2021	EPI_ISL_1469275	16/02/2021	Asia / Indonesia / East Java	B.1.466.2	GH
hCoV-19/Indonesia/JI-NIHRD-WGS01186/2021	EPI_ISL_1469240	18/02/2021	Asia / Indonesia / East Java	B.1	GH
hCoV-19/Indonesia/JI-NIHRD-WGS01185/2021	EPI_ISL_1469284	18/02/2021	Asia / Indonesia / East Java	B.1.466.2	GH
hCoV-19/Indonesia/JI-ITD-39869NTv/2021	EPI_ISL_2284878	18/02/2021	Asia / Indonesia / East Java / Surabaya	B.1.470	GH
hCoV-19/Indonesia/JI-ITD-39945NTv/2021	EPI_ISL_2284879	19/02/2021	Asia / Indonesia / East Java / Sidoarjo	B.1.470	GH
hCoV-19/Indonesia/JI-ITD-40187NTv/2021	EPI_ISL_2284880	24/02/2021	Asia / Indonesia / East Java / Surabaya	B.1.470	GH
hCoV-19/Indonesia/JI-ITD-40538NTv/2021	EPI_ISL_2284881	03/03/2021	Asia / Indonesia / East Java / Surabaya	B.1.470	GH
hCoV-19/Indonesia/JI-ITD-40544NTv/2021	EPI_ISL_2284882	03/03/2021	Asia / Indonesia / East Java / Surabaya	B.1.470	GH
hCoV-19/Indonesia/JI-ITD-40618NTv/2021	EPI_ISL_2284883	05/03/2021	Asia / Indonesia / East Java / Sidoarjo	B.1.470	GH
hCoV-19/Indonesia/JI-ITD-43154Nv/2021	EPI_ISL_2284869	09/03/2021	Asia / Indonesia / East Java / Surabaya	B.1	GH
hCoV-19/Indonesia/JI-NIHRD-WGS01832/2021	EPI_ISL_1469250	10/03/2021	Asia / Indonesia / East Java	B.1.470	GH
hCoV-19/Indonesia/JI-ITD-43155Nv/2021	EPI_ISL_2284870	10/03/2021	Asia / Indonesia / East Java / Surabaya	B.1.470	GH
hCoV-19/Indonesia/JI-ITD-43156Nv/2021	EPI_ISL_2284871	14/03/2021	Asia / Indonesia / East Java / Surabaya	B.1	GH
hCoV-19/Indonesia/JI-ITD-43158Nv/2021	EPI_ISL_2274437	15/03/2021	Asia / Indonesia / East Java / Surabaya	B.1.466.2	GH
hCoV-19/Indonesia/JI-ITD-43157Nv/2021	EPI_ISL_2284872	15/03/2021	Asia / Indonesia / East Java / Surabaya	B.1.466.2	GH
hCoV-19/Indonesia/JI-ITD-43159Nv/2021	EPI_ISL_2284884	16/03/2021	Asia / Indonesia / East Java / Surabaya	B.1.470	GH
hCoV-19/Indonesia/JI-ITD-41307NTv/2021	EPI_ISL_2274438	20/03/2021	Asia / Indonesia / East Java / Surabaya	B.1.470	GH
hCoV-19/Indonesia/JI-NIHRD-WGS02633/2021	EPI_ISL_2047570	22/03/2021	Asia / Indonesia / East Java	B.1.470	GH
hCoV-19/Indonesia/JI-ITD-43153Nv/2021	EPI_ISL_2284868	29/03/2021	Asia / Indonesia / East Java / Surabaya	B.1.466.2	GH
hCoV-19/Indonesia/JI-NIHRD-WGS02634/2021	EPI_ISL_2047571	05/04/2021	Asia / Indonesia / East Java	B.1.470	GH

hCoV-19/Indonesia/JI-ITD-42606N/2021	EPI_ISL_2226647	21/04/2021	Asia / Indonesia / East Java / Surabaya	B.1.1.7	GRY
hCoV-19/Indonesia/JI-NIHRD-WGS03205/2021	EPI_ISL_2258207	21/04/2021	Asia / Indonesia / East Java / Surabaya	B.1.1.7	GR
hCoV-19/Indonesia/JI-NIHRD-WGS03204/2021	EPI_ISL_2262277	21/04/2021	Asia / Indonesia / East Java / Surabaya	B.1.466.2	GH
hCoV-19/Indonesia/JI-NIHRD-WGS02910/2021	EPI_ISL_2854695	22/04/2021	Asia / Indonesia / East Java	B.1.466.2	GH
hCoV-19/Indonesia/JI-NIHRD-WGS02911/2021	EPI_ISL_2854696	23/04/2021	Asia / Indonesia / East Java	B.1.1.419	GR
hCoV-19/Indonesia/JI-NIHRD-WGS03202/2021	EPI_ISL_2262276	25/04/2021	Asia / Indonesia / East Java / Surabaya	B.1.466.2	GH
hCoV-19/Indonesia/JI-GS-ITD-42414N/2021	EPI_ISL_7189399	26/04/2021	Asia / Indonesia / East Java / Surabaya	B.1.470	GH
hCoV-19/Indonesia/JI-GS-ITD-42463NT/2021	EPI_ISL_7189408	26/04/2021	Asia / Indonesia / East Java / Surabaya	B.1.466.2	GH
hCoV-19/Indonesia/JI-NIHRD-WGS02909/2021	EPI_ISL_2854694	27/04/2021	Asia / Indonesia / East Java	B.1.466.2	GH
hCoV-19/Indonesia/JI-GS-ITD-42524NT/2021	EPI_ISL_7189417	28/04/2021	Asia / Indonesia / East Java / Surabaya	B.1.470	GH
hCoV-19/Indonesia/JI-NIHRD-WGS03210/2021	EPI_ISL_2854699	03/05/2021	Asia / Indonesia / East Java	B.1.466.2	GH
hCoV-19/Indonesia/JI-NIHRD-WGS03211/2021	EPI_ISL_2854700	03/05/2021	Asia / Indonesia / East Java	B.1.466.2	GH
hCoV-19/Indonesia/JI-ITD-42727N/2021	EPI_ISL_2226648	05/05/2021	Asia / Indonesia / East Java / Surabaya	B.1.351	GH
hCoV-19/Indonesia/JI-NIHRD-c.00.21.34336/2021	EPI_ISL_2617464	05/05/2021	Asia / Indonesia / East Java	B.1.466.2	GH
hCoV-19/Indonesia/JI-NIHRD-c.00.21.34337/2021	EPI_ISL_2617465	05/05/2021	Asia / Indonesia / East Java	B.1.466.2	GH
hCoV-19/Indonesia/JI-NIHRD-WGS03600/2021	EPI_ISL_2868931	05/05/2021	Asia / Indonesia / East Java	None	G
hCoV-19/Indonesia/JI-NIHRD-WGS03630/2021	EPI_ISL_3138809	06/05/2021	Asia / Indonesia / East Java / Kediri	B.1.351	GH
hCoV-19/Indonesia/JI-ITD-42745Nv/2021	EPI_ISL_4056004	07/05/2021	Asia / Indonesia / East Java / Surabaya	B.1.351	GH
hCoV-19/Indonesia/JI-ITD-42829Nv/2021	EPI_ISL_4056005	12/05/2021	Asia / Indonesia / East Java / Surabaya	B.1.351	GH
hCoV-19/Indonesia/JI-ITD-42832Nv/2021	EPI_ISL_4056006	12/05/2021	Asia / Indonesia / East Java / Surabaya	B.1.351	GH

hCoV-19/Indonesia/JI-ITD-42834Nv/2021	EPI_ISL_4056007	12/05/2021	Asia / Indonesia / East Java / Surabaya	None	G
hCoV-19/Indonesia/JI-ITD-42835Nv/2021	EPI_ISL_4056008	12/05/2021	Asia / Indonesia / East Java / Surabaya	B.1.351	GH
hCoV-19/Indonesia/JI-ITD-42831N/2021	EPI_ISL_4742352	12/05/2021	Asia / Indonesia / East Java / Surabaya	B.1.1.7	GR
hCoV-19/Indonesia/JI-NIHRD-WGS05033/2021	EPI_ISL_2987634	26/05/2021	Asia / Indonesia / East Java	AY.23	GK
hCoV-19/Indonesia/JI-GS-ITD-43249N/2021	EPI_ISL_5884798	26/05/2021	Asia / Indonesia / East Java / Surabaya	B.1.1.7	GR
hCoV-19/Indonesia/JI-ITD-43184NTv/2021	EPI_ISL_4056009	27/05/2021	Asia / Indonesia / East Java / Surabaya	B.1.466.2	GH
hCoV-19/Indonesia/JI-ITD-43356NTv/2021	EPI_ISL_3267186	28/05/2021	Asia / Indonesia / East Java / Surabaya	B.1.466.2	GH
hCoV-19/Indonesia/JI-ITD-43254NTv/2021	EPI_ISL_4056010	28/05/2021	Asia / Indonesia / East Java / Malang	B.1.466.2	GH
hCoV-19/Indonesia/JI-ITD-43285Nv/2021	EPI_ISL_3279412	31/05/2021	Asia / Indonesia / East Java / Sampang	B.1.470	GH
hCoV-19/Indonesia/JI-GS-ITD-43285N/2021	EPI_ISL_5884799	31/05/2021	Asia / Indonesia / East Java / Sampang	B.1.470	GH
hCoV-19/Indonesia/JI-ITD-43355NTv/2021	EPI_ISL_4056011	02/06/2021	Asia / Indonesia / East Java / Surabaya	B.1.466.2	GH
hCoV-19/Indonesia/JI-ITD-43358NTv/2021	EPI_ISL_4056012	02/06/2021	Asia / Indonesia / East Java / Surabaya	B.1.466.2	GH
hCoV-19/Indonesia/JI-NIHRD-WGS10118/2021	EPI_ISL_4254872	02/06/2021	Asia / Indonesia / East Java / Malang	AY.24	GK
hCoV-19/Indonesia/JI-NIHRD-WGS10119/2021	EPI_ISL_4254873	02/06/2021	Asia / Indonesia / East Java / Malang	AY.24	GK
hCoV-19/Indonesia/JI-NIHRD-WGS10120/2021	EPI_ISL_4254874	03/06/2021	Asia / Indonesia / East Java / Malang	AY.23	GK
hCoV-19/Indonesia/JI-ITD-43689N/2021	EPI_ISL_2617439	04/06/2021	Asia / Indonesia / East Java / Surabaya	AY.24	GK
hCoV-19/Indonesia/JI-NIHRD-WGS10122/2021	EPI_ISL_4254876	04/06/2021	Asia / Indonesia / East Java / Malang	AY.23	GK
hCoV-19/Indonesia/JI-GS-ITD-43466NT/2021	EPI_ISL_5884800	04/06/2021	Asia / Indonesia / East Java / Surabaya	B.1.470	GH
hCoV-19/Indonesia/JI-NIHRD-WGS10121/2021	EPI_ISL_4254875	05/06/2021	Asia / Indonesia / East Java / Malang	AY.23	GK
hCoV-19/Indonesia/JI-ITD-43589N/2021	EPI_ISL_2510687	06/06/2021	Asia / Indonesia / East Java / Surabaya	AY.24	GK
hCoV-19/Indonesia/JI-ITD-43547N/2021	EPI_ISL_2570805	06/06/2021	Asia / Indonesia / East Java / Surabaya	AY.24	GK
hCoV-19/Indonesia/JI-ITD-43550N/2021	EPI_ISL_2570806	06/06/2021	Asia / Indonesia / East Java / Surabaya	AY.24	GK
hCoV-19/Indonesia/JI-ITD-43574NT/2021	EPI_ISL_2570808	06/06/2021	Asia / Indonesia / East Java / Surabaya	AY.24	GK
hCoV-19/Indonesia/JI-ITD-43584N/2021	EPI_ISL_2570809	06/06/2021	Asia / Indonesia / East Java / Surabaya	AY.24	GK
hCoV-19/Indonesia/JI-ITD-43591N/2021	EPI_ISL_2570811	06/06/2021	Asia / Indonesia / East Java / Surabaya	AY.24	GK
hCoV-19/Indonesia/JI-ITD-43571NT/2021	EPI_ISL_2617435	06/06/2021	Asia / Indonesia / East Java / Surabaya	AY.24	GK

hCoV-19/Indonesia/JI-ITD-43575N/2021	EPI_ISL_2617436	06/06/2021	Asia / Indonesia / East Java / Surabaya	AY.24	GK
hCoV-19/Indonesia/JI-ITD-43585N/2021	EPI_ISL_2617437	06/06/2021	Asia / Indonesia / East Java / Surabaya	AY.24	GK
hCoV-19/Indonesia/JI-ITD-43595NT/2021	EPI_ISL_2617438	06/06/2021	Asia / Indonesia / East Java / Surabaya	B.1.466.2	GH
hCoV-19/Indonesia/JI-ITD-43592N/2021	EPI_ISL_2693669	06/06/2021	Asia / Indonesia / East Java / Surabaya	AY.23	GK
hCoV-19/Indonesia/JI-ITD-43576N/2021	EPI_ISL_2693670	06/06/2021	Asia / Indonesia / East Java / Surabaya	AY.24	GK
hCoV-19/Indonesia/JI-ITD-43688NT/2021	EPI_ISL_2693671	06/06/2021	Asia / Indonesia / East Java / Surabaya	AY.24	GK
hCoV-19/Indonesia/JI-NIHRD-WGS08014/2021	EPI_ISL_3138863	06/06/2021	Asia / Indonesia / East Java / Malang	AY.51	GK
hCoV-19/Indonesia/JI-NIHRD-WGS08016/2021	EPI_ISL_3138865	06/06/2021	Asia / Indonesia / East Java / Malang	AY.23	GK
hCoV-19/Indonesia/JI-NIHRD-WGS10123/2021	EPI_ISL_4254877	06/06/2021	Asia / Indonesia / East Java / Malang	AY.24	GK
hCoV-19/Indonesia/JI-ITD-43568NTv/2021	EPI_ISL_3267187	07/06/2021	Asia / Indonesia / East Java / Lamongan	AY.23	GK
hCoV-19/Indonesia/JI-ITD-43811Nv/2021	EPI_ISL_4056013	11/06/2021	Asia / Indonesia / East Java / Surabaya	AY.24	GK
hCoV-19/Indonesia/JI-ITD-43841NTv/2021	EPI_ISL_4056014	11/06/2021	Asia / Indonesia / East Java / Surabaya	AY.24	GK
hCoV-19/Indonesia/JI-GS-ITD-52548N/2021	EPI_ISL_7189629	12/06/2021	Asia / Indonesia / East Java / Surabaya	AY.24	GK
hCoV-19/Indonesia/JI-GS-ITD-52549N/2021	EPI_ISL_7189638	12/06/2021	Asia / Indonesia / East Java / Surabaya	AY.24	GK
hCoV-19/Indonesia/JI-GS-ITD-52553N/2021	EPI_ISL_7189647	12/06/2021	Asia / Indonesia / East Java / Surabaya	B.1.466.2	GH
hCoV-19/Indonesia/JI-GS-ITD-52577N/2021	EPI_ISL_7189658	12/06/2021	Asia / Indonesia / East Java / Surabaya	AY.23	GK
hCoV-19/Indonesia/JI-GS-ITD-52579N/2021	EPI_ISL_7189666	12/06/2021	Asia / Indonesia / East Java / Surabaya	AY.24	GK
hCoV-19/Indonesia/JI-GS-ITD-52583N/2021	EPI_ISL_7189674	12/06/2021	Asia / Indonesia / East Java / Surabaya	AY.24	GK
hCoV-19/Indonesia/JI-ITD-44415NTv/2021	EPI_ISL_3279414	14/06/2021	Asia / Indonesia / East Java / Malang	AY.23	GK
hCoV-19/Indonesia/JI-GS-ITD-44376NT/2021	EPI_ISL_5884801	14/06/2021	Asia / Indonesia / East Java / Situbondo	AY.24	GK
hCoV-19/Indonesia/JI-GS-ITD-44391NT/2021	EPI_ISL_5884802	14/06/2021	Asia / Indonesia / East Java / Tuban	AY.24	GK
hCoV-19/Indonesia/JI-ITD-43992Nv/2021	EPI_ISL_3279413	15/06/2021	Asia / Indonesia / East Java / Pasuruan	AY.23	GK
hCoV-19/Indonesia/JI-NIHRD-WGS10127/2021	EPI_ISL_4254880	15/06/2021	Asia / Indonesia / East Java / Malang	AY.23	GK
hCoV-19/Indonesia/JI-GS-ITD-44577NT/2021	EPI_ISL_5884804	15/06/2021	Asia / Indonesia / East Java / Kediri	AY.23	GK
hCoV-19/Indonesia/JI-ITD-44255NTv/2021	EPI_ISL_4056015	17/06/2021	Asia / Indonesia / East Java / Mojokerto	AY.24	GK
hCoV-19/Indonesia/JI-GS-ITD-51940N/2021	EPI_ISL_5884833	17/06/2021	Asia / Indonesia / East Java / Probolinggo	B.1.466.2	GH
hCoV-19/Indonesia/JI-GS-ITD-52126NT/2021	EPI_ISL_5884838	17/06/2021	Asia / Indonesia / East Java / Blitar	AY.24	GK
hCoV-19/Indonesia/JI-GS-ITD-52129NT/2021	EPI_ISL_5884839	17/06/2021	Asia / Indonesia / East Java / Blitar	AY.24	GK

hCoV-19/Indonesia/JI-GS-ITD-44571NT/2021	EPI_ISL_5884803	19/06/2021	Asia / Indonesia / East Java / Ponorogo	AY.23	GK
hCoV-19/Indonesia/JI-GS-ITD-51949N/2021	EPI_ISL_5884834	21/06/2021	Asia / Indonesia / East Java / Probolinggo	AY.24	GK
hCoV-19/Indonesia/JI-NIHRD-WGS07799/2021	EPI_ISL_4090553	22/06/2021	Asia / Indonesia / East Java / Magetan	AY.23	GK
hCoV-19/Indonesia/JI-NIHRD-WGS08015/2021	EPI_ISL_3138864	23/06/2021	Asia / Indonesia / East Java / Malang	AY.23	GK
hCoV-19/Indonesia/JI-NIHRD-WGS07798/2021	EPI_ISL_4090552	23/06/2021	Asia / Indonesia / East Java / Magetan	AY.23	GK
hCoV-19/Indonesia/JI-NIHRD-WGS07800/2021	EPI_ISL_4090554	23/06/2021	Asia / Indonesia / East Java / Magetan	AY.23	GK
hCoV-19/Indonesia/JI-GS-ITD-52125NT/2021	EPI_ISL_5884837	23/06/2021	Asia / Indonesia / East Java / Blitar	AY.24	GK
hCoV-19/Indonesia/JI-NIHRD-WGS07801/2021	EPI_ISL_4090555	24/06/2021	Asia / Indonesia / East Java / Magetan	AY.23	GK
hCoV-19/Indonesia/JI-GS-ITD-45299NT/2021	EPI_ISL_7189426	24/06/2021	Asia / Indonesia / East Java / Jombang	AY.24	GK
hCoV-19/Indonesia/JI-PNF-213674/2021	EPI_ISL_6425643	30/06/2021	Asia / Indonesia / East Java / Surabaya	AY.23	GK
hCoV-19/Indonesia/JI-PNF-213673/2021	EPI_ISL_6425645	30/06/2021	Asia / Indonesia / East Java / Surabaya	AY.23	GK
hCoV-19/Indonesia/JI-GS-ITD-46037NT/2021	EPI_ISL_5884805	05/07/2021	Asia / Indonesia / East Java / Surabaya	AY.24	GK
hCoV-19/Indonesia/JI-GS-ITD-46099NT/2021	EPI_ISL_5884806	05/07/2021	Asia / Indonesia / East Java / Gresik	AY.24	GK
hCoV-19/Indonesia/JI-GS-ITD-46107NT/2021	EPI_ISL_5884807	05/07/2021	Asia / Indonesia / East Java / Gresik	AY.23	GK
hCoV-19/Indonesia/JI-GS-ITD-52137NT/2021	EPI_ISL_7189523	07/07/2021	Asia / Indonesia / East Java / Surabaya	AY.24	GK
hCoV-19/Indonesia/JI-GS-ITD-52138NT/2021	EPI_ISL_7189529	07/07/2021	Asia / Indonesia / East Java / Trenggalek	AY.24	GK
hCoV-19/Indonesia/JI-GS-ITD-52539NT/2021	EPI_ISL_7189601	09/07/2021	Asia / Indonesia / East Java / Blitar	AY.23	GK
hCoV-19/Indonesia/JI-GS-ITD-52541NT/2021	EPI_ISL_7189610	09/07/2021	Asia / Indonesia / East Java / Blitar	AY.23	GK
hCoV-19/Indonesia/JI-GS-ITD-52542NT/2021	EPI_ISL_7189622	09/07/2021	Asia / Indonesia / East Java / Blitar	AY.23	GK
hCoV-19/Indonesia/JI-GS-ITD-46736NT/2021	EPI_ISL_5884808	12/07/2021	Asia / Indonesia / East Java / Surabaya	AY.23	GK
hCoV-19/Indonesia/JI-GS-ITD-51802NT/2021	EPI_ISL_5884816	12/07/2021	Asia / Indonesia / East Java / Madiun	AY.24	GK
hCoV-19/Indonesia/JI-GS-ITD-51803NT/2021	EPI_ISL_5884817	12/07/2021	Asia / Indonesia / East Java / Madiun	AY.24	GK
hCoV-19/Indonesia/JI-NIHRD-WGS09842/2021	EPI_ISL_4104532	13/07/2021	Asia / Indonesia / East Java / Nganjuk	B.1.466.2	GH
hCoV-19/Indonesia/JI-NIHRD-WGS09843/2021	EPI_ISL_4104533	13/07/2021	Asia / Indonesia / East Java / Nganjuk	AY.23	GK

hCoV-19/Indonesia/JI-NIHRD-WGS09844/2021	EPI_ISL_4104534	13/07/2021	Asia / Indonesia / East Java / Nganjuk	AY.24	GK
hCoV-19/Indonesia/JI-NIHRD-WGS09845/2021	EPI_ISL_4104535	13/07/2021	Asia / Indonesia / East Java / Nganjuk	AY.23	GK
hCoV-19/Indonesia/JI-NIHRD-WGS09846/2021	EPI_ISL_4104536	13/07/2021	Asia / Indonesia / East Java / Nganjuk	B.1.466.2	GH
hCoV-19/Indonesia/JI-GS-ITD-46798NT/2021	EPI_ISL_5884809	13/07/2021	Asia / Indonesia / East Java / Surabaya	AY.23	GK
hCoV-19/Indonesia/JI-GS-ITD-46885NT/2021	EPI_ISL_5884810	13/07/2021	Asia / Indonesia / East Java / Surabaya	AY.24	GK
hCoV-19/Indonesia/JI-GS-ITD-52472N/2021	EPI_ISL_7189555	13/07/2021	Asia / Indonesia / East Java / Ngawi	AY.23	GK
hCoV-19/Indonesia/JI-GS-ITD-52473NT/2021	EPI_ISL_7189564	13/07/2021	Asia / Indonesia / East Java / Ngawi	AY.23	GK
hCoV-19/Indonesia/JI-GS-ITD-52474NT/2021	EPI_ISL_7189574	13/07/2021	Asia / Indonesia / East Java / Ngawi	AY.23	GK
hCoV-19/Indonesia/JI-GS-ITD-52475NT/2021	EPI_ISL_7189579	13/07/2021	Asia / Indonesia / East Java / Ngawi	AY.23	GK
hCoV-19/Indonesia/JI-GS-ITD-52478NT/2021	EPI_ISL_7189585	13/07/2021	Asia / Indonesia / East Java / Ngawi	AY.23	GK
hCoV-19/Indonesia/JI-GS-ITD-52480NT/2021	EPI_ISL_7189593	13/07/2021	Asia / Indonesia / East Java / Ngawi	AY.23	GK
hCoV-19/Indonesia/JI-GS-ITD-51796NT/2021	EPI_ISL_5884811	16/07/2021	Asia / Indonesia / East Java / Malang	AY.23	GK
hCoV-19/Indonesia/JI-GS-ITD-51798NT/2021	EPI_ISL_5884815	16/07/2021	Asia / Indonesia / East Java / Malang	AY.23	GK
hCoV-19/Indonesia/JI-NIHRD-WGS09052/2021	EPI_ISL_4090583	18/07/2021	Asia / Indonesia / East Java / Magetan	AY.23	GK
hCoV-19/Indonesia/JI-PNF-214354/2021	EPI_ISL_6425638	21/07/2021	Asia / Indonesia / East Java / Surabaya	AY.24	GK
hCoV-19/Indonesia/JI-ITD-48475NT/2021	EPI_ISL_4056016	24/07/2021	Asia / Indonesia / East Java / Surabaya	AY.24	GK
hCoV-19/Indonesia/JI-NIHRD-WGS09847/2021	EPI_ISL_4104537	26/07/2021	Asia / Indonesia / East Java / Nganjuk	AY.23	G
hCoV-19/Indonesia/JI-ITD-48946NT/2021	EPI_ISL_4056017	28/07/2021	Asia / Indonesia / East Java / Surabaya	AY.24	GK
hCoV-19/Indonesia/JI-GS-ITD-51924N/2021	EPI_ISL_5884822	28/07/2021	Asia / Indonesia / East Java / Surabaya	AY.59	GK
hCoV-19/Indonesia/JI-GS-ITD-51925N/2021	EPI_ISL_5884823	28/07/2021	Asia / Indonesia / East Java / Surabaya	AY.79	GK
hCoV-19/Indonesia/JI-GS-ITD-51926N/2021	EPI_ISL_5884824	28/07/2021	Asia / Indonesia / East Java / Surabaya	AY.59	GK
hCoV-19/Indonesia/JI-GS-ITD-51927N/2021	EPI_ISL_5884825	28/07/2021	Asia / Indonesia / East Java / Surabaya	AY.59	GK
hCoV-19/Indonesia/JI-GS-ITD-51928N/2021	EPI_ISL_5884826	28/07/2021	Asia / Indonesia / East Java / Surabaya	AY.59	GK
hCoV-19/Indonesia/JI-GS-ITD-51929N/2021	EPI_ISL_5884827	28/07/2021	Asia / Indonesia / East Java / Surabaya	AY.59	GK
hCoV-19/Indonesia/JI-GS-ITD-51930N/2021	EPI_ISL_5884828	28/07/2021	Asia / Indonesia / East Java / Surabaya	B.1.617.2	GK
hCoV-19/Indonesia/JI-GS-ITD-51931N/2021	EPI_ISL_5884829	28/07/2021	Asia / Indonesia / East Java / Surabaya	AY.79	GK

hCoV-19/Indonesia/JI-NIHRD-WGS10126/2021	EPI_ISL_4254879	29/07/2021	Asia / Indonesia / East Java / Malang	B.1.466.2	GH
hCoV-19/Indonesia/JI-ITD-49337NT/2021	EPI_ISL_4742355	31/07/2021	Asia / Indonesia / East Java / Sidoarjo	AY.23	GK
hCoV-19/Indonesia/JI-NIHRD-WGS10094/2021	EPI_ISL_4254869	02/08/2021	Asia / Indonesia / East Java / Bondowoso	AY.23	GK
hCoV-19/Indonesia/JI-NIHRD-WGS10092/2021	EPI_ISL_4254867	03/08/2021	Asia / Indonesia / East Java / Bondowoso	AY.23	GK
hCoV-19/Indonesia/JI-NIHRD-WGS10093/2021	EPI_ISL_4254868	03/08/2021	Asia / Indonesia / East Java / Bondowoso	AY.23	GK
hCoV-19/Indonesia/JI-NIHRD-WGS10095/2021	EPI_ISL_4254870	03/08/2021	Asia / Indonesia / East Java / Bondowoso	AY.24	GK
hCoV-19/Indonesia/JI-GS-ITD-51932N/2021	EPI_ISL_5884830	05/08/2021	Asia / Indonesia / East Java / Surabaya	AY.59	GK
hCoV-19/Indonesia/JI-ITD-50088NT/2021	EPI_ISL_4056018	06/08/2021	Asia / Indonesia / East Java / Sidoarjo	AY.23	GK
hCoV-19/Indonesia/JI-NIHRD-WGS10124/2021	EPI_ISL_4254878	07/08/2021	Asia / Indonesia / East Java / Malang	AY.23	GK
hCoV-19/Indonesia/JI-GS-ITD-51922N/2021	EPI_ISL_5884821	07/08/2021	Asia / Indonesia / East Java / Surabaya	AY.59	GK
hCoV-19/Indonesia/JI-NIHRD-WGS10096/2021	EPI_ISL_4254871	09/08/2021	Asia / Indonesia / East Java / Bondowoso	AY.23	GK
hCoV-19/Indonesia/JI-GS-ITD-50826NT/2021	EPI_ISL_7189436	13/08/2021	Asia / Indonesia / East Java / Gresik	AY.23	GK
hCoV-19/Indonesia/JI-GS-ITD-51316N/2021	EPI_ISL_7189439	25/08/2021	Asia / Indonesia / East Java / Pasuruan	AY.24	GK
hCoV-19/Indonesia/JI-GS-ITD-51353NT/2021	EPI_ISL_7189448	26/08/2021	Asia / Indonesia / East Java / Surabaya	AY.23	GK
hCoV-19/Indonesia/JI-GS-ITD-51357NT/2021	EPI_ISL_7189452	26/08/2021	Asia / Indonesia / East Java / Sidoarjo	AY.24	GK
hCoV-19/Indonesia/JI-GS-ITD-51361NT/2021	EPI_ISL_7189460	26/08/2021	Asia / Indonesia / East Java / Sidoarjo	AY.23	GK
hCoV-19/Indonesia/JI-GS-ITD-51362NT/2021	EPI_ISL_7189465	26/08/2021	Asia / Indonesia / East Java / Sidoarjo	AY.23	GK
hCoV-19/Indonesia/JI-ITD-51418N/2021	EPI_ISL_4056019	28/08/2021	Asia / Indonesia / East Java / Surabaya	AY.24	GK
hCoV-19/Indonesia/JI-GS-ITD-51490NT/2021	EPI_ISL_7189468	29/08/2021	Asia / Indonesia / East Java / Surabaya	AY.24	GK
hCoV-19/Indonesia/JI-GS-ITD-51493NT/2021	EPI_ISL_7189477	30/08/2021	Asia / Indonesia / East Java / Surabaya	AY.24	GK
hCoV-19/Indonesia/JI-GS-ITD-51511NT/2021	EPI_ISL_7189482	31/08/2021	Asia / Indonesia / East Java / Surabaya	AY.23	GK
hCoV-19/Indonesia/JI-GS-ITD-51936N/2021	EPI_ISL_5884832	01/09/2021	Asia / Indonesia / East Java / Surabaya	B.1.617.2	GK
hCoV-19/Indonesia/JI-GS-ITD-51681NT/2021	EPI_ISL_7189492	03/09/2021	Asia / Indonesia / East Java / Surabaya	AY.23	GK
hCoV-19/Indonesia/JI-GS-ITD-51935N/2021	EPI_ISL_5884831	06/09/2021	Asia / Indonesia / East Java / Surabaya	B.1.617.2	GK
hCoV-19/Indonesia/JI-GS-ITD-51881N/2021	EPI_ISL_5884818	09/09/2021	Asia / Indonesia / East Java / Surabaya	AY.59	GK

hCoV-19/Indonesia/JI-GS-ITD-51882N/2021	EPI_ISL_5884819	09/09/2021	Asia / Indonesia / East Java / Surabaya	AY.23	GK
hCoV-19/Indonesia/JI-GS-ITD-51883NT/2021	EPI_ISL_5884820	10/09/2021	Asia / Indonesia / East Java / Surabaya	AY.24	GK
hCoV-19/Indonesia/JI-GS-ITD-51890NT/2021	EPI_ISL_7189503	10/09/2021	Asia / Indonesia / East Java / Surabaya	AY.23	GK
hCoV-19/Indonesia/JI-GS-ITD-51970N/2021	EPI_ISL_5884835	13/09/2021	Asia / Indonesia / East Java / Surabaya	AY.23	GK
hCoV-19/Indonesia/JI-GS-ITD-52029N/2021	EPI_ISL_5884836	13/09/2021	Asia / Indonesia / East Java / Surabaya	AY.59	GK
hCoV-19/Indonesia/JI-GS-ITD-51953NT/2021	EPI_ISL_7189510	13/09/2021	Asia / Indonesia / East Java / Surabaya	AY.23	GK
hCoV-19/Indonesia/JI-GS-ITD-52122NT/2021	EPI_ISL_7189520	21/09/2021	Asia / Indonesia / East Java / Surabaya	AY.23	GK
hCoV-19/Indonesia/JI-GS-ITD-52236NT/2021	EPI_ISL_7189541	27/09/2021	Asia / Indonesia / East Java / Surabaya	AY.23	GK
hCoV-19/Indonesia/JI-GS-ITD-53184NT/2021	EPI_ISL_8806068	06/10/2021	Asia / Indonesia / East Java / Surabaya	AY.24	GK
hCoV-19/Indonesia/JI-GS-ITD-52458N/2021	EPI_ISL_7189549	11/10/2021	Asia / Indonesia / East Java / Surabaya	AY.23	GK
hCoV-19/Indonesia/JI-GS-ITD-53186NT/2021	EPI_ISL_8806069	11/10/2021	Asia / Indonesia / East Java / Surabaya	AY.23	GK
hCoV-19/Indonesia/JI-GS-ITD-52728NT/2021	EPI_ISL_7189724	21/10/2021	Asia / Indonesia / East Java / Blitar	AY.23	GK
hCoV-19/Indonesia/JI-GS-ITD-53189NT/2021	EPI_ISL_8806070	21/10/2021	Asia / Indonesia / East Java / Surabaya	AY.23	GK
hCoV-19/Indonesia/JI-GS-ITD-52650NT/2021	EPI_ISL_7189680	23/10/2021	Asia / Indonesia / East Java / Surabaya	AY.79	GK
hCoV-19/Indonesia/JI-GS-ITD-52671NT/2021	EPI_ISL_7189687	23/10/2021	Asia / Indonesia / East Java / Surabaya	AY.79	GK
hCoV-19/Indonesia/JI-GS-ITD-52673NT/2021	EPI_ISL_7189698	23/10/2021	Asia / Indonesia / East Java / Surabaya	AY.79	GK
hCoV-19/Indonesia/JI-GS-ITD-52729NT/2021	EPI_ISL_7189730	23/10/2021	Asia / Indonesia / East Java / Blitar	AY.23	GK
hCoV-19/Indonesia/JI-GS-ITD-52736NT/2021	EPI_ISL_7189737	23/10/2021	Asia / Indonesia / East Java / Blitar	AY.23	GK
hCoV-19/Indonesia/JI-GS-ITD-53191NT/2021	EPI_ISL_8806079	26/10/2021	Asia / Indonesia / East Java / Surabaya	AY.100	GK
hCoV-19/Indonesia/JI-GS-ITD-52718N/2021	EPI_ISL_7189705	30/10/2021	Asia / Indonesia / East Java / Surabaya	AY.24	GK
hCoV-19/Indonesia/JI-GS-ITD-52719N/2021	EPI_ISL_7189714	30/10/2021	Asia / Indonesia / East Java / Surabaya	AY.24	GK
hCoV-19/Indonesia/JI-GS-ITD-53193NT/2021	EPI_ISL_8806074	30/10/2021	Asia / Indonesia / East Java / Surabaya	AY.24	GK
hCoV-19/Indonesia/JI-GS-ITD-52956NT/2021	EPI_ISL_8215780	12/11/2021	Asia / Indonesia / East Java / Surabaya	AY.23	GK
hCoV-19/Indonesia/JI-GS-ITD-52776N/2021	EPI_ISL_8215777	16/11/2021	Asia / Indonesia / East Java / Surabaya	AY.23	GK
hCoV-19/Indonesia/JI-GS-ITD-52958N/2021	EPI_ISL_8215781	18/11/2021	Asia / Indonesia / East Java / Surabaya	AY.23	GK
hCoV-19/Indonesia/JI-GS-ITD-52959N/2021	EPI_ISL_8215782	18/11/2021	Asia / Indonesia / East Java / Surabaya	AY.23	GK
hCoV-19/Indonesia/JI-GS-ITD-52960N/2021	EPI_ISL_8215783	19/11/2021	Asia / Indonesia / East Java / Surabaya	AY.23	GK
hCoV-19/Indonesia/JI-NIHRD-WGS13534/2021	EPI_ISL_7550151	20/11/2021	Asia / Indonesia / East Java	AY.24	GK
hCoV-19/Indonesia/JI-GS-ITD-52962N/2021	EPI_ISL_8215784	25/11/2021	Asia / Indonesia / East Java / Surabaya	AY.23	GK
hCoV-19/Indonesia/JI-GS-ITD-52847N/2021	EPI_ISL_8215778	26/11/2021	Asia / Indonesia / East Java / Surabaya	AY.23	GK
hCoV-19/Indonesia/JI-GS-ITD-52963N/2021	EPI_ISL_8215785	26/11/2021	Asia / Indonesia / East Java / Surabaya	AY.23	GK

hCoV-19/Indonesia/JI-GS-ITD-53195NT/2021	EPI_ISL_8806084	26/11/2021	Asia / Indonesia / East Java / Surabaya	AY.24	GV
hCoV-19/Indonesia/JI-GS-ITD-52953NT/2021	EPI_ISL_8216465	28/11/2021	Asia / Indonesia / East Java / Ponorogo	AY.100	GK
hCoV-19/Indonesia/JI-GS-ITD-53011NT/2021	EPI_ISL_8215791	29/11/2021	Asia / Indonesia / East Java / Surabaya	AY.23	GK
hCoV-19/Indonesia/JI-GS-ITD-53012NT/2021	EPI_ISL_8215792	29/11/2021	Asia / Indonesia / East Java / Surabaya	AY.23	GK
hCoV-19/Indonesia/JI-GS-ITD-52954NT/2021	EPI_ISL_8215779	30/11/2021	Asia / Indonesia / East Java / Ponorogo	AY.23	GK
hCoV-19/Indonesia/JI-GS-ITD-53014NT/2021	EPI_ISL_8215793	30/11/2021	Asia / Indonesia / East Java / Surabaya	AY.23	GK
hCoV-19/Indonesia/JI-GS-ITD-52953NT/2021	EPI_ISL_8216465	28/11/2021	Asia / Indonesia / East Java / Ponorogo	AY.100	GK
hCoV-19/Indonesia/JI-GS-ITD-53011NT/2021	EPI_ISL_8215791	29/11/2021	Asia / Indonesia / East Java / Surabaya	AY.23	GK
hCoV-19/Indonesia/JI-GS-ITD-53012NT/2021	EPI_ISL_8215792	29/11/2021	Asia / Indonesia / East Java / Surabaya	AY.23	GK
hCoV-19/Indonesia/JI-GS-ITD-52954NT/2021	EPI_ISL_8215779	30/11/2021	Asia / Indonesia / East Java / Ponorogo	AY.23	GK
hCoV-19/Indonesia/JI-GS-ITD-53014NT/2021	EPI_ISL_8215793	30/11/2021	Asia / Indonesia / East Java / Surabaya	AY.23	GK
hCoV-19/Indonesia/JI-GS-ITD-52953NT/2021	EPI_ISL_8216465	28/11/2021	Asia / Indonesia / East Java / Ponorogo	AY.100	GK
hCoV-19/Indonesia/JI-GS-ITD-53011NT/2021	EPI_ISL_8215791	29/11/2021	Asia / Indonesia / East Java / Surabaya	AY.23	GK
hCoV-19/Indonesia/JI-GS-ITD-53012NT/2021	EPI_ISL_8215792	29/11/2021	Asia / Indonesia / East Java / Surabaya	AY.23	GK
hCoV-19/Indonesia/JI-GS-ITD-52954NT/2021	EPI_ISL_8215779	30/11/2021	Asia / Indonesia / East Java / Ponorogo	AY.23	GK
hCoV-19/Indonesia/JI-GS-ITD-53014NT/2021	EPI_ISL_8215793	30/11/2021	Asia / Indonesia / East Java / Surabaya	AY.23	GK
hCoV-19/Indonesia/JI-GS-ITD-52953NT/2021	EPI_ISL_8216465	28/11/2021	Asia / Indonesia / East Java / Ponorogo	AY.100	GK
hCoV-19/Indonesia/JI-GS-ITD-53011NT/2021	EPI_ISL_8215791	29/11/2021	Asia / Indonesia / East Java / Surabaya	AY.23	GK
